# Application of response surface methodology and quantitative NMR for the optimum extraction, characterization, and quantitation of *Antrodia cinnamomea* triterpenoids

**DOI:** 10.1038/s41598-023-47615-6

**Published:** 2023-11-20

**Authors:** Chi-Ying Li, Yang-Chang Wu, Fang-Rong Chang, Mohamed El-Shazly, Ying-Chi Du, Chi-Yu Lu, Tsai-Hui Duh, Tung-Ying Wu

**Affiliations:** 1 Graduate Institute of Natural Products, College of Pharmacy, Kaohsiung Medical University, Kaohsiung, 807 Taiwan; 2 Department of Pharmacology and Pharmaceutical Sciences, School of Pharmacy, University of Southern California, 1985 Zonal Avenue, Los Angeles, CA 90089 USA; 3 Graduate Institute of Integrated Medicine, China Medical University, Taichung, 404 Taiwan; 4 Chinese Medicine Research and Development Center, China Medical University Hospital, Taichung, 404 Taiwan; 5 Drug Development and Value Creation Research Center, Kaohsiung Medical University, Kaohsiung, 807 Taiwan; 6 Department of Medical Research, Kaohsiung Medical University Hospital, Kaohsiung Medical University, Kaohsiung, 807 Taiwan; 7 Department of Marine Biotechnology and Resources, National Sun Yat-sen University, Kaohsiung, 804 Taiwan; 8 Department of Pharmacognosy, Faculty of Pharmacy, Ain-Shams University, Organization of African Unity Street, Abassia, Cairo, 11566 Egypt; 9 Department of Biochemistry, College of Medicine, Kaohsiung Medical University, Kaohsiung, 807 Taiwan; 10 Department of Medicinal and Applied Chemistry, Kaohsiung Medical University, Kaohsiung, 807 Taiwan; 11 Research Center for Precision Environmental Medicine, Kaohsiung Medical University, Kaohsiung, 807 Taiwan; 12 Department of Biological Science and Technology, Meiho University, Pingtung, 912 Taiwan; 13 Department of Food Science and Nutrition, Meiho University, Pingtung, 912 Taiwan

**Keywords:** Analytical chemistry, Organic chemistry, Scientific data, Chemistry

## Abstract

*Antrodia cinnamomea* (AC) is a treasured Asian medicinal mushroom, which has attracted attention due to recent research on its effectiveness in targeting a variety of serious ailments such as cancer and liver diseases. Among different *A. cinnamomea* constituents, triterpenoids are regarded as the most therapeutically attractive components because of their anti-inflammatory and cytotoxic activities. In the present study, we proposed a mathematical and statistical extraction protocol to evaluate the concentrations of total ergostane and lanostane triterpenoid derivatives from the ethanolic extract of the wild fruiting bodies of *A. cinnamomea* (EEAC) by utilizing response surface methodology (RSM) and quantitative NMR (qNMR) approaches. The optimum response surface model showed that the variations of the investigated response variables reached more than 90%, suggesting that the developed model is accurate in explaining response variability. Furthermore, the EEAC major characteristic triterpenoids were quantified through the comparison of the HPLC-tandem MS results with those of the qNMR results. The precision of the used techniques was also evaluated. The experimental design of the EEAC optimum extraction procedure obtained by using RSM and qNMR enabled accurate characterization and quantitation of *A. cinnamomea* triterpenoids.

## Introduction

*Antrodia cinnamomea* (AC), also known as *Antrodia camphorata* or *Taiwanofungus camphoratus*, is a precious medicinal mushroom native to Taiwan and is renowned for its fascinating pharmacological activities including anti-inflammation, anti-cancer, immunomodulatory, and hepato-protective effects^1–5^. It has also been used as folk medicine by the Taiwanese aboriginal tribes for the treatment of various health-related issues such as food and alcohol detoxification, abdominal pain, diarrhea, and hypertension, and to improve liver function^1^. Several classes of chemical constituents were purified and identified from *A. cinnamomea* such as benzenoids, terpenoids, benzoquinone, lignans, polysaccharides, maleic and succinic acid derivatives^2–4^. Among them, the terpenoids, the main bioactive components of *A. cinnamomea* fruiting bodies, demonstrated many fascinating pharmacological effects^4^. *A. cinnamomea* contains complicated tetracyclic triterpenoids including ergostane-type and lanostane-type triterpenoids^2, 4^. These types of triterpenoids show similar chemical structures resulting in technical difficulties in their separation for quality control purposes or the preparation of commercial products^6^.

Highly efficient extraction and analytical methodologies were developed for the purification and quantification of biologically active secondary metabolites. However, few reports are available on the quantification of triterpenoids, especially ergostanes and lanostanes derivatives, and their use as chemical markers for the quality control of *A. cinnamomea*. Several research groups reported the use of the colorimetric method with the vanillin/acetic acid system to quantify AC total triterpenoids^7, 8^. However, colorimetric method protocols suffered from low sensitivity and poor accuracy in the determination of the concentrations of AC total triterpenoids. Currently, there are no reference standard protocols for the quality control of AC active constituents from different AC materials. Thus, developing proper standardization and an efficient quality control protocol to identify the concentration of bioactive components will provide necessary information on the quality and biological activity of AC commercial products. AC triterpenoids are intriguing chemical markers for AC products that are present in major quantities, which can be quantified and inspected by spectroscopic techniques.

Previously, we developed and established a feasible quantification methodology to comprehensively evaluate the quality of benzenoid components of AC wild fruiting bodies and their related commercial cultivation products^9^. Additionally, we developed a convenient fractionation process to concentrate the bioactive triterpenoids from AC fruiting bodies. The chemical profile of the triterpenoids-rich fraction was revealed by HPLC, HPLC–PDA/MS, and NMR strategies^10^. In the current study, we established and developed a mathematical and statistical protocol to evaluate the concentrations of the total ergostane and lanostane triterpenoid derivatives, from the ethanolic extract of the wild fruiting bodies of AC (EEAC) using response surface methodology (RSM) and quantitative NMR (qNMR). The total triterpenoids in EEAC were quantified using the HPLC-tandem MS methodology.

## Materials and methods

### Standard compounds and AC materials

**Figure 1:**
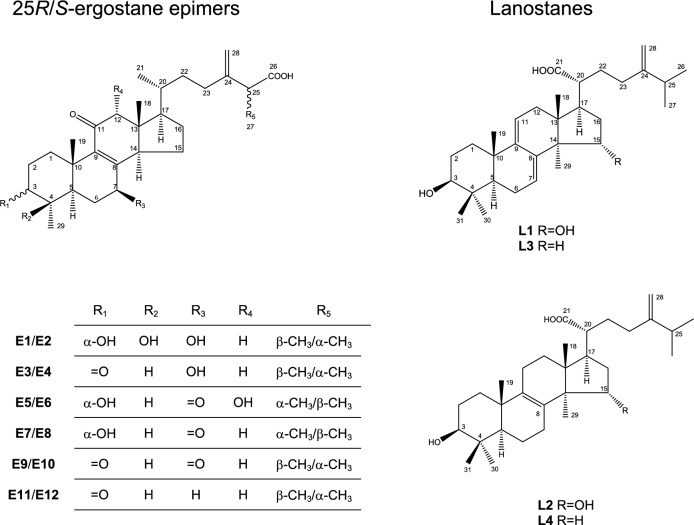
The ergostane epimers and lanostane triterpenoids.

The standard compounds of 25*R*/*S*-ergostane epimers triterpenoids (**E1**–**E12**) and lanostane triterpenoids (**L1**–**L4**) employed for comparative analysis experiments were separated from the triterpenoid-rich fraction (FEA) of EEAC. They were 25*S*/*R*-antcin K (**E1**/**E2**), 25*S*/*R*-antcin C (**E3**/**E4**), 25*R*/*S*-zhankuic acid C (**E5**/**E6**), 25*R*/*S*-zhankuic acid B (**E7**/**E8**), 25*S*/*R*-zhankuic acid A (**E9**/**E10**) and 25*S*/*R*-antcin A (**E11**/**E12**) of ergostane epimers triterpenoids and dehydrosulphurenic acid (**L1**), sulphurenic acid (**L2**), dehydroeburicoic acid (**L3**) and eburicoic acid (**L4**) of lanostane triterpenoids (Fig. 1). The structures of sixteen isolates were confirmed and elucidated based on NMR and MS data as illustrated in our previous study and the results were compared with those reported in the literature (Fig. S1)^4, 10^. The used internal standard (IS), ganoderic acid A, for tandem MS experiment was isolated in our laboratory. The internal standard, pyrazine, used for quantitative NMR analysis was purchased from Sigma-Aldrich (St. Louis, MO, USA). The purity of these analytes was higher than 97%, which was evaluated with a three-point peak purity method and was determined using Shimadzu “Class VP” software.

The wild fruiting bodies of *A. cinnamomea* were collected from the forest in Taiwan in 2020 and was further identified on the basis of the morphological characteristics and nucleotide sequence of the internal transcribed spacers (ITS) analysis using universal primers by Professor Tun-Tschu Chang (Taiwan Forestry Research Institute). The obtained sequences of ribosomal RNA/ITS was further blasted with the National Center for Biotechnology Information (NCBI) database. The blasting results showed that the sample shared the 99.5% sequence identity with *A. cinnamomea* (GenBank accession number: AY378095). The cutting wood culture (CWC) and dish culture (DC) samples were purchased from different Taiwan Biotech Co., Ltd in 2022. The voucher specimens were deposited in the authors' laboratory.

### Determination and quantification of the EEAC total ergostane and lanostane triterpenoids with ^1^H NMR spectrum

The determination and quantification of the total ergostane and lanostane triterpenoids in EEAC were performed using the NMR spectrum analysis method. The NMR apparatus used was Varian UNITY plus 400 MHz spectrometers, and each sample was examined 10 times on the basis of the following parameters: the spectra width was established to 6002.4 Hz, and the width of the impulse intensity was established to 6.3 µs. The EEAC was added to the internal standard solution (0.132 mg pyrazine dissloved in 0.6 mL DMSO-*d6*). In the detection analysis of the total ergostane and lanostane triterpenoids, the starting and ending points of the characteristic proton signals were manually selected to calculate the integration for every peak, which were the terminal olefinic proton signals (H-28) at *δ*_H_ 4.82 (2H, br d) of ergostane triterpenoid and signals at *δ*_H_ 4.63 (1H, s) and 4.70 (1H, s) of lanostane triterpenoid, respectively. The ratios of the characteristic proton integration area to its corresponding integration area of the added internal standard were calculated. In the quantitative analysis, the standard compounds of ergostane and lanostane triterpenoids with different concentrations were prepared to establish a specific calibration plot under investigation, which were zhankuic acid A (**E9**/**E10**) of the ergostane triterpenoid in the range of 2.02 to 6.06 mg and dehydroeburicoic acid (**L3**) of the lanostane triterpenoid in the range of 1.15 to 5.01 mg, respectively. All experiments were accomplished in triplicate, and the relative standard deviation was calculated. The absolute concentrations of the total ergostane and lanostane triterpenoids in EEAC were obtained by introducing the obtained ratio to the calibration curve equation below:

$${Y}_{E}=11.8 {X}_{E}+3.5, {R}^{2}=0.997$$

$${Y}_{L}=11.0 {X}_{L}+1.1, {R}^{2}=0.999$$

where ${Y}_{E}$ and ${Y}_{L}$ are the ergostane and lanostane triterpenoids ratios of the characteristic proton integration area of the standard compounds to its corresponding integration area of the additional internal standard in EEAC. ${X}_{E}$ and ${X}_{L}$ are the absolute concentrations of the total ergostane and lanostane triterpenoids in EEAC.

### EEAC preparation procedures

The ethanolic extract of AC wild fruiting bodies (EEAC) was prepared as described previously^11^. In brief, the mushroom samples were refluxed with ethanol in a 1:10 (w/v) ratio at 75 °C for 2 h, and the ethanolic extracts were then cooled and enabled to precipitate at 4 °C overnight. The supernatant of the extracts was filtrated to remove the precipitate, yielding EEAC that was stored at 0–4 °C before analysis. Following the establishment and development of "Green Chemistry" principles over the past few decades, environmentally friendly methods are becoming more attractive. Therefore, developing an efficient extraction and eco-friendly protocol that reduces the use of large quantities of the slow growing and expensive AC will be highly desirable. To compare the extraction efficiency of different protocols, the ultrasonic-assisted extraction (UAE) method was selected to evaluate the concentrations of the total ergostane and lanostane triterpenoids from EEAC in the preliminary test. The UAE experiment was carried out using an ultrasonic apparatus with a working frequency of 40 kHz and the power of 150 W (Delta DC150H, Taiwan). The bath was a temperature-controlled rectangular container (300 × 160 × 150 mm, 7.2 L). A set of 50 ml Erlenmeyer flasks was filled with 2.5 g of ground AC samples of 3 mm particle size (controlled with a ratio of powder (g): ethanol (ml) of 1:10) and ultrasonicated in an ultrasonic bath with a working frequency of 40 kHz at 75 °C for 2 h.

The extraction conditions of UAE were the same as those mentioned above and the concentrations of the total ergostane and lanostane triterpenoids were obtained by calculating their integrations area of the characteristic proton signals to its corresponding integrations area of the added internal standard in the ^1^H NMR spectra. The results indicated that the integrations of the total ergostane and lanostane triterpenoids from EEAC obtained using the UAE method (39.98 and 12.03) were similar to those obtained by the reflux mode (40.01 and 12.11). Furthermore, in our preliminary test, the EEAC yield of the UAE extraction method was similar to those measured by the reflux mode in the range of 29–31% at 75 ºC for 2 h. Therefore, the UAE method can be used in the present study as an extraction procedure for the quantitative analysis of EEAC because of its high reproducibility, shorter time for extraction, simple procedures, reduced solvent consumption, lower temperature, and lower energy input.

### Experimental design of EEAC extraction

The optimization parameters of the UAE extraction of the total ergostane and lanostane triterpenoids from EEAC were conducted using an on face experimental strategy based on a three factor/three level design known as a rotatable central composite design. The application of a CCD design is a convenient tool for optimizing a procedure with three levels (−1, 0, and + 1) for every factor. The purpose of this design was performed to evaluate the effects and interactions of three independent variables, namely temperature (°C, ${\rm X}1$), time (minutes, ${\rm X}2$) and ethanol concentrations (EtOH%, ethanol–water ratio, v/v, ${\rm X}3$). Three levels of values for the independent variables were selected and demonstrated in their coded and uncoded forms based on the preliminary test (Table 1). A set of designs of experiments consisting of sixteen independent runs, which include six replicates of a central point, each designated by the coded value 0, were selected in randomized order based on a CCD-On face configuration for three factors.

**Table 1:** Central composite design setting in the coded and uncoded form of the independent variables (${\rm X}_{1}$, ${\rm X}_{2}$ and ${\rm X}_{3}$) along with the experimental response.

Run	Coded and uncoded variable levels			Integration ratios ($Y$)^a^			
	${\rm X}_{1}$/temperature	${\rm X}_{2}$/exaction time	${\rm X}_{3}$/EtOH proportion	Ergostane (actual values)	Lanostane (actual values	Ergostane (predicted values)	Lanostane (predicted values)
1	25 (−1)	30 (−1)	35 (−1)	27.58	9.63	27.89	9.57
2	25 (−1)	30 (−1)	95 (1)	37.18	11.81	36.71	11.72
3	25 (−1)	60 (0)	65 (0)	34.52	11.43	34.67	11.58
4	25 (−1)	90 (1)	35 (−1)	26.94	9.74	26.90	9.78
5	25 (−1)	90 (1)	95 (1)	36.81	12.07	36.85	12.02
6	50 (0)	30 (−1)	65 (0)	35.56	12.64	35.01	12.56
7	50 (0)	60 (0)	35 (−1)	30.20	11.89	28.77	11.54
8	50 (0)	60 (0)	65 (0)	34.31	12.81	35.88	12.93
9	50 (0)	60 (0)	65 (0)	34.57	12.17	35.88	12.93
10	50 (0)	60 (0)	95 (1)	40.33	13.59	40.31	13.49
11	50 (0)	90 (1)	65 (0)	35.15	12.87	34.26	12.51
12	75 (1)	30 (−1)	35 (−1)	26.38	10.67	26.70	10.83
13	75 (1)	30 (−1)	95 (1)	39.43	12.42	39.83	12.49
14	75 (1)	60 (0)	65 (0)	36.91	12.88	35.31	12.29
15	75 (1)	90 (1)	35 (−1)	24.22	10.22	25.06	10.42
16	75 (1)	90 (1)	95 (1)	39.28	12.01	39.33	12.18

^a^Integration ratios: the total ergostane and lanostane triterpenoids integration ratios of the characteristic proton integration area to its corresponding integration area of the added internal standard in EEAC.

### Statistical and mathematical analysis

Statistical analysis was performed using Design Expert software JMP (Version 9.0.0, 2010 SAS Institute Inc.). The response of the UAE extraction procedure, the ergostane and lanostane triterpenoids ratios of the characteristic proton integration area of the standard compounds to the corresponding integration area of the added internal standard ($Y$) was modeled as a second-order polynomial equation. This equation enclosed a function of the entire primary variables and their interactions as displayed in the following equation.

$$Y= {\beta }_{0}+{\sum }_{i=1}^{n}{\beta }_{i}{X}_{i}+{\sum }_{i=1}^{n}{\beta }_{ii}{X}_{i}^{2}+{\sum }_{1\le i\le j}^{n}{\beta }_{ij}{X}_{i}{X}_{j}$$

where ${X}_{i}$ and ${X}_{j}$ are the coded variables, ${\beta }_{0}$, ${\beta }_{i}$, ${\beta }_{ii}$ and ${\beta }_{ij}$ are the model coefficient for intercept, first-order, quadratic, and interaction between ${X}_{i}$ and ${X}_{j}$, respectively. The model obtained from the regression analysis was used to generate the response surface and contour plots. The adequacy of the model was determined by evaluating the lack of fit, coefficient of determination (*R*^2^), and the Fisher test value (F-value) acquired from the analysis of variance (ANOVA) which was obtained from the software. Significant variables were those with a confidence level higher than 95% (*p* < 0.05). The validation of the model illustrated that the optimal conditions for the extraction of the total ergostane and lanostane triterpenoids from EEAC, depending on the extraction temperature, time and ethanol–water ratio were obtained using the predictive RSM equations.

To generate response surfaces, the software used the quadratic model equation shown above. Three-dimensional response surface plots and contour plots were created by retaining one response variable at its optimum levels and plotting it against two factors (independent variables). For each solvent, response surface plots were generated. Additionally, the coded values of the experimental factors and factor levels utilized for the response surface analysis are illustrated in Table 1. The entire design using aqueous EtOH and water as extraction solvents consisted of 16 experimental points including six replications of the center point. The coded values for the experimental designs are shown in Table 1.

### HPLC and HPLC-tandem MS conditions for EEAC analysis

The analysis of EEAC was performed using an LC-20A VP HPLC system (Shimadzu Inc.) equipped with a quaternary solvent pump (LC-20AT), an autosampler (SIL-20AD), an online degasser (DGU-14A), a photodiode-array detector (SPD-M20A) and the Class VP software for data collection. An Agilent Poroshell 120 EC-C_18_ column (150 mm × 4.6 mm, i.d., 2.7 µm, Agilent Technologies, CA, USA) was used for liquid chromatography. The injection volume of the sample was 10 µL. The optimum mobile phase is composed of H_2_O containing 0.1% formic acid and 3 mM ammonium acetate (A) and ACN (B). A gradient program was used as follows, the preliminary elution condition was A–B (61:39, v/v), which was linearly changed to A–B (56:44, v/v) at 15 min, A–B (55:45, v/v) at 17.5 min, A–B (53:47, v/v) at 22.5 min, A–B (50:50, v/v) at 27.5 min, A–B (47:53, v/v) at 30 min, A–B (45:55, v/v) at 35 min, A–B (35:65, v/v) at 45 min, A–B (2:98, v/v) at 55 min, then increased to 100% B in 5 min, and eventually decreased to 39% B in 1 min and held for 9 min, for regeneration. The mobile phase was filtered through a 0.22 µm Millipore filter and was degassed before use. The flow rate was adjusted to 1.3 mL/min, the column temperature was maintained at ambient temperature, and the detection wavelength was performed at 254 nm. Before loading to the HPLC column, 1 mg of the EEAC dry extract was dissolved in 1 mL of methanol and was further filtered through the 0.45 µm membrane filter.

The quantitative HPLC-tandem mass experiment was carried out using an Agilent 1200 HPLC system (Agilent Technologies) equipped with an API 4000 triple quadrupole mass spectrometer (Applied Biosystem, CA, USA). The ion source for the detection of electrospray ionization was selected as the negative ion mode and the analytical conditions were similar to those described previously. The quantification was accomplished in multiple reactions monitoring (MRM) mode using the precursor-to-product ion transitions of 487 to 443, 469 to 425, 485 to 441, 469 to 425, 467 to 423 and 453 to 409, corresponding to 25*R*/*S*-ergostane epimers triterpenoids (**E1**–**E12**) and 483 to 83, 485 to 355, 467 to 337, 469 to 339 and 515 to 497, corresponding to lanostane triterpenoid (**L1**–**L4**) and IS, respectively. The optimized ESI source parameters were as follows, the ion spray voltage is −4500 V, the nitrogen nebulizer gas pressure is 60 psi, the nitrogen auxiliary gas pressure is 55 psi, the nitrogen curtain gas pressure is 12 psi, the heater temperature is 600 °C, and collisionally activated dissociation (CAD) gas is 12. To maximize the intensity of the transition ions, the cone voltage and collision energy were optimized for each tested sample. The detailed information of specific declustering potential (DP), collision energies (CE), collision cell exit potential (CXP), and MRM transitions of **E1**–**E12**, **L1**–**L4,** and IS are given in Table 2. All data acquisition and processing were accomplished using Analyst 1.5.1 software (AB SCIEX, Concord, ON, Canada).

**Table 2:** Compounds dependent parameters and the calibration curve data of sixteen analytes and IS (ganoderic acid A) in MRM mode for HPLC-tandem MS analysis.

Analytes	Retention time (*t*_R_)	Parent ions [M − H]^−^	Daughter ions (*m/z*)	DP	CE	CXP	Calibration curve equation	*r*^2^
E1	3.96	487	443^a^/407	−91 −91	−31 −39	−10 −8	*Y* = (0.00408 ± 0.00006)*x* −(0.00477 ± 0.00437)	0.997
E2	4.23	487	443^a^/407	−90 −90	−32 −39	−7 −11	*Y* = (0.00396 ± 0.00016)*x* + (0.00748 ± 0.00523)	0.999
E3	17.10	469	425^a^/247	−66 −66	−32 −43	−18 −8	*Y* = (0.01550 ± 0.00091)*x* −(0.18615 ± 0.04059)	0.998
E4	18.70	469	425^a^/247	−66 −66	−32 −43	−11 −14	*Y* = (0.00976 ± 0.00039)*x* −(0.12398 ± 0.02426)	0.997
E5	19.88	485	441^a^/413	−75 −75	−31 −37	−8 −11	*Y* = (0.00697 ± 0.00009)*x* + (0.05383 ± 0.02665)	0.997
E6	20.53	485	441^a^/413	−89 −89	−32 −36	−10 −11	*Y* = (0.00629 ± 0.00010)*x* −(0.02017 ± 0.00685)	0.998
E7	29.43	469	425^a^/409	−78 −78	−30 −52	−11 −8	*Y* = (0.00122 ± 0.00001)*x* −(0.02136 ± 0.00626)	0.998
E8	29.87	469	425^a^/409	−70 −70	−31 −51	−10 −12	*Y* = (0.00147 ± 0.00011)*x* −(0.02376 ± 0.01168)	0.997
E9	32.23	467	423^a^/407	−80 −80	−31 −42	−11 −9	*Y* = (0.02541 ± 0.00031)*x* + (0.19959 ± 0.15536)	0.998
E10	32.85	467	423^a^/407	−77 −77	−31 −50	−11 −11	*Y* = (0.03640 ± 0.00173)*x* −(0.22022 ± 0.11636)	0.999
E11	42.29	453	409^a^/393	−70 −70	−31 −48	−15 −7	*Y* = (0.00069 ± 0.00002)*x* −(0.00934 ± 0.00062)	0.999
E12	42.64	453	409^a^/393	−66 −66	−32 −50	−11 −8	*Y* = (0.00051 ± 0.00001)*x* −(0.01150 ± 0.00134)	0.999
L1	28.83	483	268/83^a^	−135 −135	−48 −62	−7 −7	*Y* = (0.00070 ± 0.00002)*x* −(0.00718 ± 0.00375)	0.997
L2	29.86	485	355^a^/83	−160 −160	−50 −64	−15 −11	*Y* = (0.00023 ± 0.00002)*x* + (0.00614 ± 0.00116)	0.997
L3	55.70	467	371/337^a^	−114 −114	−50 −50	−10 −9	*Y* = (0.00744 ± 0.00042)*x* + (0.14245 ± 0.12241)	0.997
L4	56.46	469	373/339^a^	−160 −160	−48 −50	−13 −7	*Y* = (0.00169 ± 0.00012)*x* + (0.00628 ± 0.00410)	0.998
IS	4.29	515	497^a^/285	−106 −106	−38 −58	−11 −12		

*DP* declustering potential, *CE* collision energy, *CXP* collision cell exit potential.

^a^Quantification transitions.

### HPLC-tandem MS calibration

To create a calibration plot for each compound under the current study, a stock solution of each standard compound (1 mg/mL in methanol) was prepared and serially diluted to yield the reference solution at various concentrations (10 to 1000 ng/mL). Each reference standard solution received an adequate amount of the internal standard (IS) (ganoderic acid A) to achieve a final concentration of 100 ng/mL. Calibration curves were generated by comparing the peak area of each compound to the IS peak area in triplicate for every concentration.

## Results and discussion

### NMR analysis of the EEAC ergostane and lanostane triterpenoids

Developing an accurate and efficient method to determine the concentrations of target active ingredients in natural products and nutritional supplements became an attractive point to researchers, particularly for the simultaneous determination of various target components with diversified physicochemical characteristics against extremely complicated matrices^12^. Thus, the identification of specific chemical markers in natural products and nutritional supplements has emerged as a straightforward quality control strategy. In general, NMR is applied to elucidate the structure of chemical compounds and biological substances. Moreover, it can be utilized to provide rapid and accurate information on the concentrations of specific constituent in a mixture. This technique is referred to quantitative NMR (qNMR), and it has found distinctive applications in biomedical, pharmaceutical, and phytochemical research^13, 14^. While compared to those other instrumental analysis techniques, qNMR is a primary ratio method because the acquired peak areas are proportional to the number of corresponding nuclei^15^. Therefore, qNMR was selected for the quantitative analysis of the EEAC total ergostane and lanostane triterpenoids due to its being considered superior to other conventional chromatographic methods for the determination of the concentrations of certain herbal mixture constituents. In our previous studies, we successfully developed efficient analytical procedures using ^1^H NMR to evaluate AC fractions^10^. The ^1^H NMR spectra of FNH (the benzenoid-rich fraction), FEA (the triterpenoid-rich fraction), FET (the saccharide-rich fraction) and EEAC under identical conditions (10 mg/0.75 ml, pyridine-*d*5, 400 MHz) were plotted for comparison. EEAC ^1^H NMR spectrum showed signals at *δ*_H_ 0.70 and 1.61, corresponding to the two tertiary methyl signals and three secondary methyl signals at *δ*_H_ 0.89, 1.03 and 1.52. Three methine signals at *δ*_H_ 3.01, 3.17 and 3.48 as well as the terminal olefinic protons (2H-28) in a 24-exo-methylene-26-oic acid sidechain at *δ*_H_ 4.89, 4.94, 5.08 and 5.24 were also detected. These distinguishable signals are the characteristic signals of AC triterpenoids, which were also observed in the ^1^H NMR spectra of FEA but were absent from the ^1^H NMR spectra of FNH and FET.

**Figure 2:**
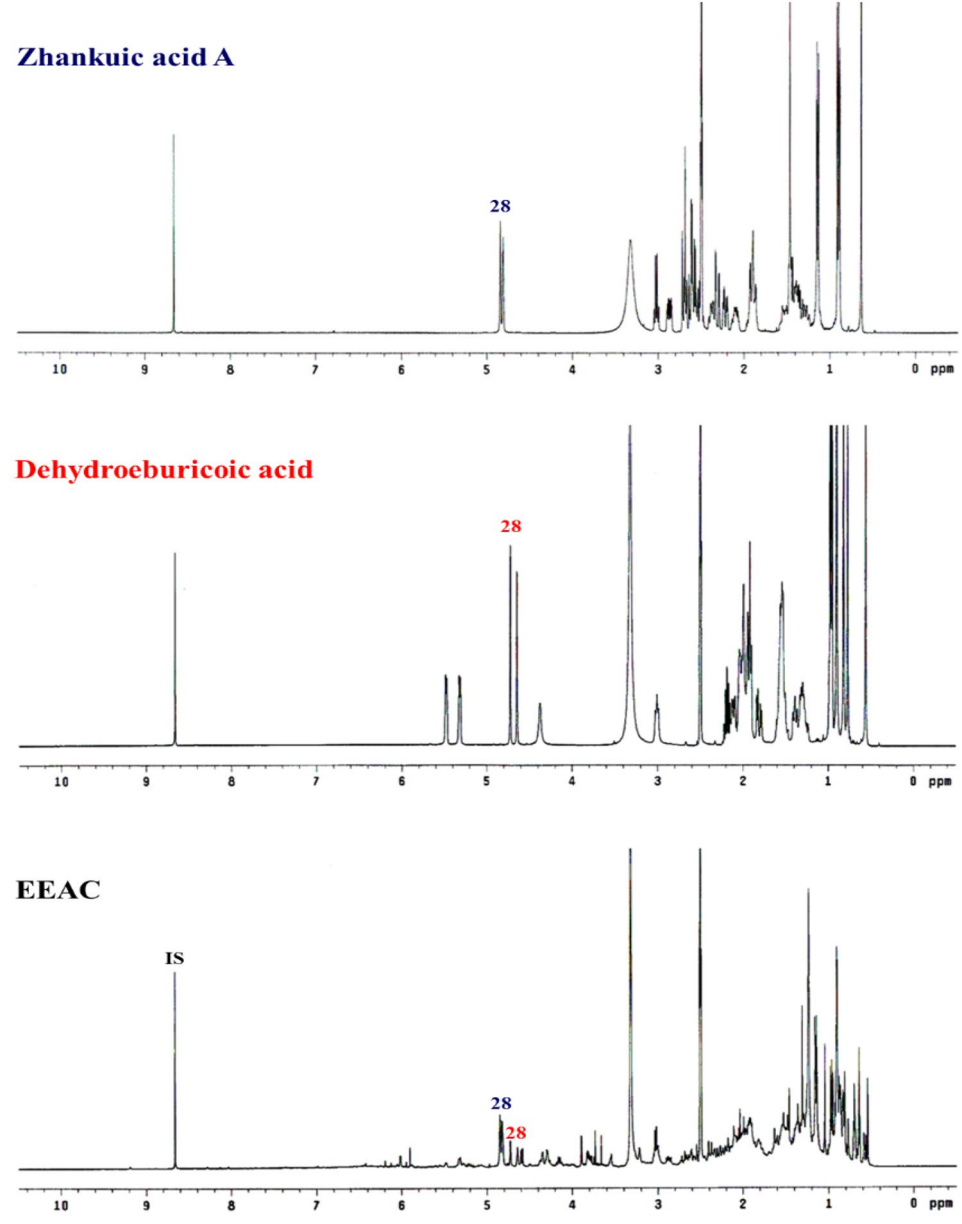
Plot and comparison of ^1^H NMR profiles of EEAC, zhankuic acid A, and dehydroeburicoic acid (400 MHz).

To provide an accurate method to evaluate the concentrations of EEAC total ergostane and lanostane triterpenoids in the present study, an efficient determination procedure was proposed utilizing ^1^H NMR. While compared to pyridine-*d*5, the polar properties of EEAC suggested that DMSO-*d*6 would be the best deuterated solvent for ensuring the solubility of all components and that its signals were not interfering with the characteristic signals of the analyte. On the other hand, the characteristic signals must be designed by integrating a well-separated specific proton signal and the signal not interfering with the deuterated solvent. For the present experiment, the terminal olefinic proton signals (H-28) of ergostane triterpenoid (2H, br d, *δ*_H_ 4.82) and the signals of lanostane triterpenoid (1H, s, *δ*_H_ 4.63) and (1H, s, *δ*_H_ 4.70) were selected. These signals were well-separated without any interference from other signals and hence their integration values would provide characterized information on the concentration of the total ergostane and lanostane triterpenoids in EEAC (Fig. 2). These distinguishable signals were also detected in the ^1^H NMR spectra of the standard compounds of zhankuic acid A (**E9**/**E10**) and dehydroeburicoic acid (**L3**) under identical conditions (10 mg dissolved in 0.6 mL DMSO-*d*6, 400 MHz). Additionally, a highly stable pure internal standard with signals that do not interfere with the characteristic signals of the analyte must be employed in the qNMR experiment. For this purpose, pyrazine, with a proton signal at *δ*_H_ 8.60, was selected as an internal standard. Moreover, zhankuic acid A and dehydroeburicoic acid as the standard compounds proved that the process developed in the present study could determine the concentrations of EEAC total ergostane and lanostane triterpenoids in the ^1^H NMR spectrum. The developed quantitative method combined the ratios of the characteristic proton integration area to its corresponding integration area of the added internal standard and the constructional calibration curves of the standard compounds can be utilized to evaluate the absolute concentrations of the total ergostane and lanostane triterpenoids in EEAC.

### Selection of extraction parameters in preliminary experiments

The extraction efficiency varies with the process conditions. For instance, some of the most important factors affecting the extraction efficiency in terms of quality and yield are temperature, contact time, and material/solvent ratio. However, the role of each factor in the mass transfer of the procedure is unpredictable because of the chemical characteristics of the solvent and the diversity of its structure as well as the composition of the natural products, which cause each material–solvent system to behave differently.

Preliminary studies were carried out to establish the optimal extraction parameters for the extraction of the total ergostane and lanostane triterpenoids from AC. The three independent variables of the solid-to-liquid (material/EtOH, w/v) ratio, time, temperature, and ethanol concentrations (EtOH%, ethanol–water ratio, v/v) were determined. The response was evaluated by calculating the total ergostane and lanostane triterpenoids integration ratios of the characteristic proton integration area of the standard compounds to its corresponding integration area of the added internal standard using the constructed calibration curves of the standard compounds in ^1^H NMR analysis. To determine the required parameters of the solid-to-liquid ratio (material/EtOH, w/v) for the extraction of the total ergostane and lanostane triterpenoids from AC, three parameters of the solid-to-liquid ratio (1:10, 1:20, and 1:30) were assessed. The results indicated that the extraction of the AC ergostane and lanostane triterpenoids was dependent on the solid-to-liquid ratio. The integration ratios of the total ergostane and lanostane triterpenoids increased with an increasing solid-to-liquid ratio, reaching a plateau at a solid-to-liquid ratio of 1:20. Thus, the solid-to-liquid ratio of 1:20 was selected. This ratio was suggested to enhance the extraction efficiency in comparison with the solid-to-liquid ratio of 1:10. The selection of an appropriate extraction parameter including time, temperature and ethanol concentrations (EtOH%) depended on our previously reported extraction condition^10^. The extraction procedure was carried out at time intervals of 30, 60, 90, and 120 min. The other extraction parameters were as follows including an ultrasonic temperature of 25 °C and a 1:20 solid-to-liquid ratio. While the extraction time varied from 30 to 90 min, the variance of the integration ratios of the total ergostane and lanostane triterpenoids were not significantly increased and reached a maximum at 60 min. This suggested that the extraction time of 30–90 min was sufficient to obtain AC ergostane and lanostane triterpenoids. Further, we tested the effect of changing the extraction temperature by experimenting at 25, 50, and 75 ºC. The other extraction parameters were as follows including an extraction time of 30 min and a 1:20 solid-to-liquid ratio. As expected, the integration ratios of the total ergostane and lanostane triterpenoids increased with the temperature and reached their maximum at 75 °C. The increase in temperature may improve the extraction efficiency by enhancing the solubility of triterpenoids, increasing the diffusion coefficient, and decreasing the viscosity coefficient. By increasing the temperature from 25 to 75 °C, the integration ratios of the total ergostane and lanostane triterpenoids increased 5.7 and 4.9%, respectively. Therefore, the extraction temperature range of 25–75 °C was optimal in the present experiment. The other parameter of ethanol concentrations (EtOH%) was suggested to be crucial in determining the efficiency of extracting AC triterpenoids due to their lipophilic properties. The extraction procedure was performed with various ethanol concentrations of 15, 35, 65, and 95% (v/v), as well as following extraction parameters: an ultrasonic temperature of 25 °C, extraction time of 30 min, and a solid-to-liquid ratio of 1:20. The results showed that the integration ratios of the total ergostane and lanostane triterpenoids increased with the increase in the ethanol concentrations. When ethanol concentrations varied from 35 to 95%, the variance of the integration ratios of the total ergostane and lanostane triterpenoids was significantly increased and reached a plateau of 95%. While the ethanol concentration increased from 35 to 95%, the integration ratios of the total ergostane and lanostane triterpenoids increased by 25.8% and 18.4%, respectively. Thus, the ethanol concentration of 35%-95% was favorable for extracting AC triterpenoids. The designed extraction parameters of the three independent variables with three levels of values were evaluated using predictive RSM equations.

### Optimization of the extraction condition of AC total ergostane and lanostane triterpenoids by RSM analysis

The experimental design matrix and the corresponding results are illustrated according to their design parameters (Table 1). The response of the integration ratios of the total ergostane and lanostane triterpenoids were recorded in the range of 24.22–40.33 and 9.63–13.59, respectively. The adequacy of the second-order quadratic equation model developed from multiple regression analysis for this experiment could be validated using the analysis of variance (ANOVA) (Table 3). For the design model, the coefficient of determination, *R*^2^ was recorded at 0.9710 of ergostane and 0.9350 of lanostane which explained the good fit of the model to the data input. For a 95% confidence interval, *P* > *F* values must be smaller than 0.05 to be considered statistically significant toward the effect of the response. In the design model, the *P* > *F* values of ergostane and lanostane were less than 0.05 suggesting that this model was significant. The significance of the model was also evaluated by the lack-of-fit test. The *P* > *F* values were more than 0.05 (insignificant) in both ergostane and lanostane implying that the model was sufficiently accurate for predicting those response variations. Using mathematical models, the significance of the investigated factors and their interactions were evaluated. The quadratic model showed that the linear variable of EtOH% (${\rm X}3$) was significant (*P* < 0.01) in both of ergostane and lanostane. The one quadratic term of temperature (${\rm X}_{1}^{2}$) was significant (*P* < 0.05) of lanostane triterpenoids (Table 4).

**Table 3:** ANOVA analysis results obtained by central composite design.

Source	Ergostane				Lanostane			
	DF	Sum of squares	*F* ratio	Prob > *F*	DF	Sum of squares	*F* ratio	Prob > *F*
${\rm X}_{1}$(25,75)	1	1.01761	0.5458	0.4879	1	1.23904	5.515	0.0572
${\rm X}_{2}$(30,90)	1	1.39129	0.7463	0.4208	1	0.00676	0.0301	0.868
${\rm X}_{3}$(35,95)	1	333.04441	178.6439	< .0001	1	9.50625	42.3126	0.0006
${\rm X}_{1}$*${\rm X}_{2}$	1	0.21125	0.1133	0.7479	1	0.1891125	0.8417	0.3943
${\rm X}_{1}$*${\rm X}_{3}$	1	9.3312	5.0052	0.0666	1	0.1176125	0.5235	0.4966
${\rm X}_{2}$*${\rm X}_{3}$	1	0.6498	0.3486	0.5765	1	0.0045125	0.0201	0.8919
${\rm X}_{1}$*${\rm X}_{1}$	1	2.07937	1.1154	0.3316	1	2.6182132	11.6537	0.0143
${\rm X}_{2}$*${\rm X}_{2}$	1	4.10683	2.2029	0.1883	1	0.4145768	1.8453	0.2232
${\rm X}_{3}$*${\rm X}_{3}$	1	4.72046	2.532	0.1627	1	0.4465336	1.9875	0.2083
ANOVA								
Model	9	375.04354	22.3524	0.0006^a^	9	19.40214	9.5955	0.0062^a^
Error	6	11.18576			6	1.348004		
C. total	15	386.22929			15	20.750144		
Lack of fit								
Lack of fit	5	11.151956	65.9879	0.0932^b^	5	1.1432039	1.1164	0.6126^b^
Pure error	1	0.0338			1	0.2048		
Total error	6	11.185756			6	1.3480039		
Summary of fit								
* R*^2^	0.971039				0.935036			
* R*^2^-adj	0.927596				0.837591			

^a^*P* > *F* values < 0.05 indicates statistical significance.

^b^*P* > *F* values > 0.05 indicates statistical insignificance.

**Table 4:** Regression coefficients of the predicted quadratic model to the responses of the total integration ratios of EEAC ergostane and lanostane triterpenoids.

Term^a^	Ergostane		Lanostane	
	Regression coefficients	*p*-values	Regression coefficients	*p*-values
Intercept	35.882069	< .0001^b^	12.931034	< .0001^b^
${\rm X}_{1}$(25,75)	0.319	0.4879	0.352	0.0572
${\rm X}_{2}$(30,90)	−0.373	0.4208	−0.026	0.868
${\rm X}_{3}$(35,95)	5.771	< .0001^b^	0.975	0.0006^b^
${\rm X}_{1}$*${\rm X}_{2}$	−0.1625	0.7479	−0.15375	0.3943
${\rm X}_{1}$*${\rm X}_{3}$	1.08	0.0666	−0.12125	0.4966
${\rm X}_{2}$*${\rm X}_{3}$	0.285	0.5765	0.02375	0.8919
${\rm X}_{1}$*${\rm X}_{1}$	−0.888103	0.3316	−0.996552	0.0143^b^
${\rm X}_{2}$*${\rm X}_{2}$	−1.248103	0.1883	−0.396552	0.2232
${\rm X}_{3}$*${\rm X}_{3}$	−1.338103	0.1627	−0.411552	0.2083

^a^Temperature (${\rm X}_{1}$), Time (${\rm X}_{2}$) and EtOH % (${\rm X}_{3}$).

^b^*P* < 0.05 indicates statistical significance.

**Figure 3:**
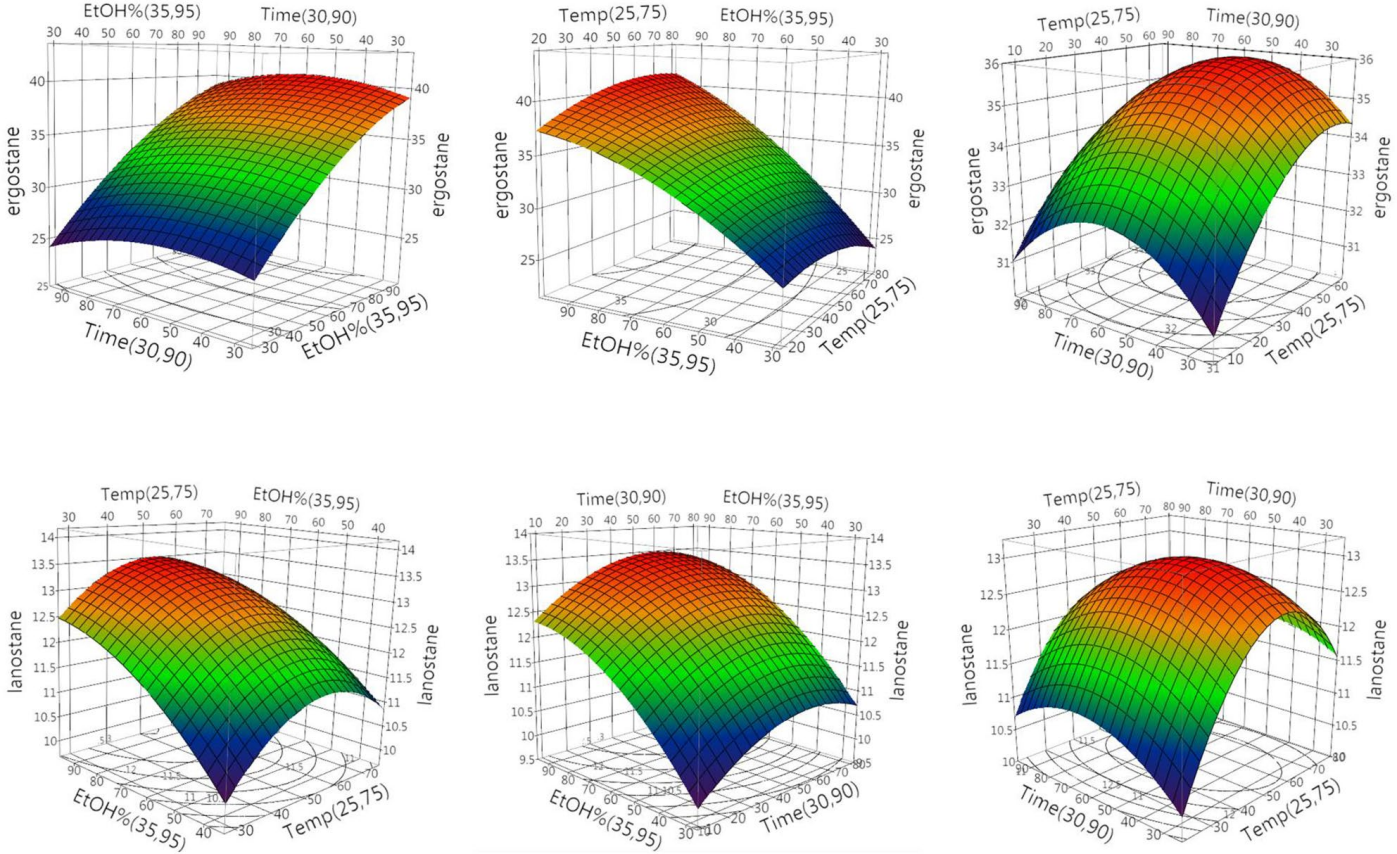
The three-dimensional response surface profiles of the integration area ratios of EEAC total ergostane and lanostane triterpenoids affected by three independent variables.

**Figure 4:**
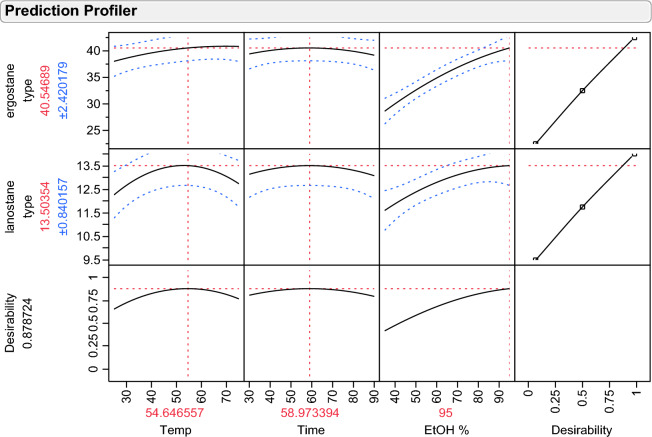
The prediction profile of the optimized extraction conditions of each factor utilizing the central composite design.

The three-dimensional representation of the response surfaces model demonstrated the relationship between the independent and dependent variables (Fig. 3). The suitability of the model equation in predicting the optimum response values of three independent variables was evaluated using these three-dimensional plots and their respective contour plots. The results indicated that the optimized conditions were the extraction temperature of 54.6 °C, the extraction time of 58.9 min, and the ethanol proportion of 95%. These conditions resulted in corresponding maximum integration ratios of the total ergostane and lanostane triterpenoids of 40.53 and 13.50, respectively (Fig. 4). However, the variation in the extraction temperature (significant, *P* < 0.05) may play a key role for the extraction AC ergostane and lanostane triterpenoids in this designed model. The discovery of an optimal extraction temperature of 54.6 °C was kind of surprising as we had anticipated that higher temperatures would enhance extraction efficiency. However, higher temperatures than this specific point had the opposite effect and reduced the yield of compounds of the lanostane type. Therefore, to obtain the highest yield of AC total triterpenoids, it was suggested to apply the optimum extraction conditions in any future experiment.

Using a combination of the developed extraction protocol and constructional calibration curves of the standard compounds, the concentrations of the EEAC total ergostane and lanostane triterpenoids were determined to be 513 ± 0.18 and 187 ± 0.25 µg/mg, respectively. The optimum response surface extraction protocol and qNMR approaches were applied to the analysis of AC total triterpenoids in different cultivation products. The developed protocol results in the concentrations of the EEAC total ergostane and lanostane triterpenoids were determined to be 292 ± 0.45 and 344 ± 0.35 µg/mg of the CWC sample and 37 ± 0.01 and 54 ± 0.03 of the DC sample, respectively. The results indicated that the media conditions and culture techniques have an important effect on fungal metabolites, resulting in products with inconsistent biological activity and concentration of major compounds.

### Characterization of the EEAC major ergostane and lanostane triterpenoids with HPLC-tandem MS

Over the last few years, several research groups reported the application of HPLC/UPLC-UV or PDA (photodiode array) detector and SFC (supercritical fluid chromatography) coupled with MS detector to analyze and quantify the components of AC fruiting bodies and mycelia^16, 17^. Despite the success of the developed methods in identifying or separating certain complicated ingredients in AC extracts, they were inefficient in quantifying AC triterpenoids. Owning to the poor sensitivity and poor accuracy of the reported methods, the desired peaks could not be analyzed simultaneously. To address this issue, a highly sensitive and selective method based on HPLC coupled with triple quadrupole mass spectrometry in multiple reactions monitoring (MRM) mode was developed to quantify the EEAC characteristic triterpenoids (**E1**–**E12** and **L1**–**L4**). The quantification of EEAC total triterpenoids obtained using RSM and qNMR was evaluated by comparing the obtained results with those collected from HPLC-tandem MS.

In our previous study, we tried to separate AC triterpenoids by evaluating the use of three different 0.1% organic acids, including trifluoroacetic, acetic or formic acids as an additive to the mobile phase. The addition of organic acids aimed to facilitate the separation of AC triterpenoids due to their acidic nature. The results suggested that the optimum mobile phase was acetonitrile containing 0.1% aqueous acetic acid, however, the major 25*R*/*S*-epimers of ergostane triterpenoids were not completely separated in the chromatogram^10^. To efficiently separate these epimers, the use of ammonium acetate at different concentrations was evaluated as an additive to the mobile phase (containing 0.1% formic acid). The results in the HPLC–UV chromatogram showed that the best resolution to separate the major ergostane epimers was a mobile phase containing 0.1% formic acid, 3 mM ammonium acetate, and acetonitrile at a detection wavelength of 254 nm (Fig. S2). Although lanostane triterpenoids (**L1**/**L2** and **L3**/**L4**) contain Δ^7,9^ orΔ^8^ double bonds, they could not be separated under these optimum conditions. To identify and quantify their concentrations, a triple quadrupole mass spectrometer was selected due to the difference in molecular weights of these analytes. An internal standard was required to accurately determine of the concentrations of triterpenoids. Particularly, an internal standard should be a stable isotope-labeled compound or a structurally related analog. However, stable isotope-labeled substances are difficult to obtain and must be handled under special precautions^18, 19^. Therefore, a structurally similar analog such as ganoderic acid A, which was isolated in our laboratory, was used in the proposed method. This analog possesses comparable solubility, chromatographic and spectrometric characteristics to the target analyte and thus can be employed as an internal standard. Initially, **E1**–**E12**, **L1**–**L4,** and the internal standard were ionized using both positive and negative ion modes through mass spectrometry. The results indicated that the negative mode was suitable and was selected for the subsequent analysis. To monitor the effect of the ionization source temperature on the MS peak intensity, three different temperatures (450 °C, 550 °C, and 600 °C) was tested. The results suggested that the optimum temperature for the ionization source was 600 °C.

The negative ESI–MS/MS spectra for the EEAC characteristic triterpenoids (**E1**–**E12** and **L1**–**L4**) are shown in Fig. S3. All investigated ergostanes in the present study possessed a COOH group at C-25, which could generate [M-44]^-^ ions from [M − H]^−^ in their *MS/MS* spectra. The loss of the fragment ion was assigned to the CO_2_ cleavage of the side chain. The fragment ions of 4-OH ergostanes of 25*S*/*R*-antcin K (**E1**/**E2**) were *m/z* 443, 425, and 407, which appeared due to the loss of CO_2_ and H_2_O. The abundant fragments of 7-OH ergostanes of 25*S*/*R*-antcin C (**E3**/**E4**) were *m*/*z* 301, 259, and 247 after the collision, and the collision energy can trigger B-ring RDA fragmentation and can lead to B/C-ring cleavage (Fig. S3). Therefore, the fragments at *m*/*z* 301, 259, and 247 could be regarded as diagnostic ions for 7-OH ergostanes, and these unique fragments were also found in other 7-OH compounds 25*S*/*R*-antcin K (**E1**/**E2**). The structure in ergostanes of 25*R*/*S*-zhankuic acid C (**E5**/**E6**) was substituted for the hydroxyl group at the C-12 position. Their *MS/MS* spectra revealed three major product ions at *m*/*z* 423, 413, and 383 fragments implying the lost ions were [M-CO_2_-H_2_O]^−^, [M-CO_2_-CO]^−^ and [M-CO_2_-CO-CH_2_O]^−^, respectively. On the other hand, the MS/MS spectra of 25*R*/*S*-zhankuic acid B (**E7**/**E8**), 25*S*/*R*-zhankuic acid A (**E9**/**E10**) and 25*S*/*R*-antcin A (**E11**/**E12**) were dominated by [M-60]^−^ ions. The loss of the fragment ion was assigned to C_2_H_4_O_2_ cleavage from the side chain (Fig. S3). The abundant [M-60]^−^ ion could be a diagnostic signal for 7-ΔO/7-H ergostanes. For the analysis of lanostanes (**L1**/**L2** and **L3**/**L4**), we combined Q1 and Q3 scans to increase their sensitivity and selected the reliable fragment ions to quantify in EEAC because of their low level of oxygenation. These fragment ions contained *m*/*z* 83 (C_6_H_11_) or *m*/*z* 97 (C_7_H_13_) from the side chain. The calibration curves were generated by plotting the peak area ratios of **E1**–**E12**, **L1**–**L4,** and the internal standard against the known concentration of each compound. The plot was revealed to be linear towards the range of 10–1000 ng/mL with *r*^2^ values of > 0.99 (Table 2).

### HPLC-tandem MS method validation and quantification of the EEAC ergostane and lanostane triterpenoids

The developed quantitative method based on the characteristic fragmentation reactions of triterpenoid compounds was highly specific with no interfering peaks at the retention times of the marker compounds (**E1**–**E12** and **L1**–**L4**). The intra-day accuracy and the precision were calculated by analyzing of characteristic triterpenoids (**E1**–**E12** and **L1**–**L4**) in five replicates at three different concentrations (50, 500 and 1000 ng/ml) on the same day. The inter-day accuracy and precision were obtained by repeating the measurements on five consecutive days. The relative standard deviation and relative error were less than 8.84, 8.14% and 8.22, 8.02% of ergostane and lanostane triterpenoids, respectively (Tables S1 and S2). The quantification of the sixteen triterpenoids was accomplished using HPLC-tandem MS protocol, the EEAC extraction method was prepared with our previous extraction procedure (refluxed with 95% ethanol at 75 °C in a 1:10 (w/v) ratio for 2 h) aiming to evaluate the accuracy of evaluating EEAC total triterpenoids concentrations using the optimum extraction conditions. The quantification results of EEAC total triterpenoids in two different extraction procedures were also compared. Therefore, the total concentrations of the ergostane and lanostane triterpenoids in the EEAC were found to be 567.83 and 113.86 µg/mg, respectively. The concentrations of each of the sixteen triterpenoids were found to be 16.739 ± 0.38, 34.539 ± 0.31 µg/mg (**E1**/**E2**), 5.997 ± 0.30, 10.751 ± 0.32 μg/mg (**E3**/**E4**), 40.206 ± 0.35, 54.288 ± 0.30 μg/mg (**E5**/**E6**), 48.102 ± 0.45, 39.012 ± 0.40 μg/mg (**E7**/**E8**), 31.509 ± 0.25, 22.251 ± 0.21 μg/mg (**E9**/**E10**), 120.418 ± 0.53, and 144.023 ± 0.57 μg/mg (**E11**/**E12**) for ergostanes and 45.685 ± 0.40, 38.561 ± 0.35, 26.724 ± 0.38, and 2.897 ± 0.41 μg/mg (**L1–L4**) for lanostane, respectively.

The results demonstrated that the optimum extraction procedures resulted in high yields of the total triterpenoids in EEAC. The concentration of the total ergostane triterpenoids in EEAC under the expected temperature (54.6 °C) achieved a plateau point. The concentration can slowly increase with increasing temperature. On the contrary, the concentration of the total lanostane triterpenoids in EEAC obtained using the optimum extraction temperature (54.6 °C) was 1.65 folds higher than those obtained by the original extraction temperature (75 °C). Consequently, the extraction temperature was an important variable for extracting the EEAC ergostane and lanostane triterpenoids in addition to the main parameter of EtOH concentration.

## Conclusions

An efficient methodology was developed for the characterization and quantification of AC triterpenoids using experimental design. The wild fruiting body of AC (EEAC) was analyzed in an accurate protocol using RSM and qNMR. The quantification of the total triterpenoids was conducted by comparing the HPLC-tandem MS and qNMR results. The lack of standard protocols to ensure AC product quality highlighted the importance of developing and establishing a quantification method to provide AC components. This method will have practical applications for the production of highly specialized AC supplements and related commercial products.

### Supplementary Information

Supplementary Information.

## Data Availability

The data that support the findings of this study are available from the corresponding author upon reasonable request.

## Abbreviations

AC: *Antrodia cinnamomea*
EEAC: Ethanolic extract from *A. cinnamomea*
FEA: The ethyl acetate fraction from EEAC (the triterpenoid-rich fraction)
UAE: Ultrasonic-assisted extraction
HPLC-PDA: High-performance liquid chromatography equipped with a photodiode array detector
HPLC-tandem MS: High-performance liquid chromatography equipped to triple quadrupole mass spectrometry
qNMR: Quantitative nuclear magnetic resonance

## References

[1] Lu MC (2013). Recent research and development of *Antrodia*
*cinnamomea*. Pharmacol. Ther..

[2] Qiao X (2015). Comprehensive chemical analysis of triterpenoids and polysaccharides in the medicinal mushroom *Antrodia*
*cinnamomea*. RSC Adv..

[3] Ganesan N, Baskaran R, Velmurugan BK, Thanh NC (2019). *Antrodia*
*cinnamomea*—An updated minireview of its bioactive components and biological activity. J. Food Biochem..

[4] Kuang Y, Li B, Wang Z, Qiao X, Ye M (2021). Terpenoids from the medicinal mushroom *Antrodia*
*camphorata*: Chemistry and medicinal potential. Nat. Prod. Rep..

[5] Zhang BB (2019). Production of bioactive metabolites by submerged fermentation of the medicinal mushroom *Antrodia*
*cinnamomea*: Recent advances and future development. Crit. Rev. Biotechnol..

[6] Xu C, Wang B, Pu Y, Tao J, Zhang T (2018). Techniques for the analysis of pentacyclic triterpenoids in medicinal plants. J. Sep. Sci..

[7] He YC, He KZ, Pu Q, Li J, Zhao ZJ (2012). Optimization of cultivating conditions for triterpenoids production from *Antrodia*
*cinnmomea*. Indian J. Microbiol..

[8] Ma TW, Lai Y, Yang FC (2014). Enhanced production of triterpenoid in submerged cultures of *Antrodia*
*cinnamomea* with the addition of citrus peel extract. Bioprocess Biosyst. Eng..

[9] Wu TY (2013). New approach to the characterization and quantification of *Antrodia*
*cinnamomea* benzenoid components utilizing HPLC-PDA, qNMR and HPLC-tandem MS: Comparing the wild fruiting bodies and its artificial cultivated commercial products. Food Res. Int..

[10] Du YC (2012). Chemical profiling of the cytotoxic triterpenoid-concentrating fraction and characterization of ergostane stereo-isomer ingredients from *Antrodia*
*camphorata*. J. Pharm. Biomed. Anal..

[11] Lu MC (2009). Active extracts of wild fruiting bodies of *Antrodia*
*camphorata* (EEAC) induce leukemia HL 60 cells apoptosis partially through histone hypoacetylation and synergistically promote anticancer effect of trichostatin A. Arch. Toxicol..

[12] Li YG, Song L, Liu M, Hu ZB, Wang ZT (2009). Advancement in analysis of *Salviae*
*miltiorrhizae* Radix et Rhizoma (Danshen). J. Chromatogr. A.

[13] Lynch MJ (1994). Ultra high field NMR spectroscopic studies on human seminal fluid, seminal vesicle and prostatic secretions. J. Pharm. Biomed. Anal..

[14] Bollard ME, Stanley EG, Lindon JC, Nicholson JK, Holmes E (2005). NMR-based metabonomic approaches for evaluating physiological influences on biofluid composition. NMR Biomed..

[15] Rundlof T (2010). Survey and qualification of internal standards for quantification by ^1^H NMR spectroscopy. J. Pharm. Biomed. Anal..

[16] Huang CH (2010). Fruiting body of Niuchangchih (*Antrodia*
*camphorata*) protects livers against chronic alcohol consumption damage. J. Agric. Food Chem..

[17] Lin TY (2011). Metabolite profiles for *Antrodia*
*cinnamomea* fruiting bodies harvested at different culture ages and from different wood substrates. J. Agric. Food Chem..

[18] Schwarz CV (2009). Developing a learning progression for scientific modeling: Making scientific modeling accessible and meaningful for learners. J. Res. Sci. Teach..

[19] Puiggros F, Sola R, Blade C, Salvado MJ, Arola L (2011). Nutritional biomarkers and foodomic methodologies for qualitative and quantitative analysis of bioactive ingredients in dietary intervention studies. J. Chromatogr. A.

