# Supplementary material for: Application of response surface methodology and quantitative NMR for the optimum extraction, characterization, and quantitation of Antrodia cinnamomea triterpenoids
Source: Sci Rep. 2023 Nov 20;13:20265. doi: 10.1038/s41598-023-47615-6 (PMC10661979; doi:10.1038/s41598-023-47615-6)
Supplement: Supplementary file 1 — Supplementary Information. [file 41598_2023_47615_MOESM1_ESM.docx]

**Supplementary information**

**Application of response surface methodology and quantitative NMR for the optimum extraction, characterization, and quantitation of *Antrodia cinnamomea* triterpenoids**

Chi-Ying Li ^1, 2^, Yang-Chang Wu ^3, 4^, Fang-Rong Chang ^1, 5, 6, 7^, Mohamed El-Shazly ^8^, Ying-Chi Du ^1^, Chi-Yu Lu ^9^, Tsai-Hui Duh ^6, 10, 13,^ *, and Tung-Ying Wu ^11, 12,^ *

*^1^ Graduate Institute of Natural Products, College of Pharmacy, Kaohsiung Medical University, Kaohsiung 807, Taiwan*

*^2^ Department of Pharmacology and Pharmaceutical Sciences, School of Pharmacy, University of Southern California, 1985 Zonal Avenue, Los Angeles, California 90089, USA*

*^3^ Graduate Institute of Integrated Medicine, China Medical University, Taichung 404, Taiwan*

*^4^ Chinese Medicine Research and Development Center, China Medical University Hospital, Taichung 404, Taiwan*

*^5^ Drug Development and Value Creation Research Center, Kaohsiung Medical University, Kaohsiung 807, Taiwan*

*^6^ Department of Medical Research, Kaohsiung Medical University Hospital, Kaohsiung Medical University, Kaohsiung 807, Taiwan*

*^7^ Department of Marine Biotechnology and Resources, National Sun Yat-sen University, Kaohsiung 804, Taiwan*

*^8^ Department of Pharmacognosy, Faculty of Pharmacy, Ain-Shams University, Organization of African Unity Street, Abassia, Cairo 11566, Egypt*

*^9^ Department of Biochemistry, College of Medicine, Kaohsiung Medical University, Kaohsiung 807, Taiwan*

*^10^ Department of Medicinal and Applied Chemistry, Kaohsiung Medical University, Kaohsiung 807, Taiwan*

*^11^ Department of Biological Science & Technology, Meiho University, Pingtung 912, Taiwan*

*^12^ Department of Food Science and Nutrition, Meiho University, Pingtung 912, Taiwan*

*^13^ Research Center for Precision Environmental Medicine, Kaohsiung Medical University, Kaohsiung 807, Taiwan*

*Corresponding authors:

Asst. Prof. Tsai-Hui Duh, Tel: +886-7-3121101-2682; Fax: +886-7-3114773; E-mail address: tshudu@kmu.edu.tw

Asst. Prof. Tung-Ying Wu, Tel: +886-8-779-9821 (ext. 8754); Fax: +886-8-779-3281; E-mail address: x00011700@meiho.edu.tw; kuma0401@gmail.com

**Contents**

**Figure S1.** The ^1^H-NMR spectrum (400 MHz) of the major characteristic ergostane and lanostane triterpenoids measured in C_5_D_5_N------------------------**S5**

**Figure S2.** An optimum condition and best resolution for separating the major ergostane compounds (**E1**-**E12**) in HPLC analysis ---------------------------------**S10**

**Figure S3.** The negative ESI-MS/MS spectra of 25R/S-ergostane epimers (**E1**-**E12**) and lanostanes (**L1**-**L4**) -------------------------------------------------------------**S11**

**Table S1.** Precision and accuracy in determination of ergostane triterpenoids (**E1**-**E12**) using HPLC-tandem MS analysis (MRM mode) ------------------------**S16**

**Table S2.** Precision and accuracy in determination of lanostane triterpenoids (**L1**-**L4**) using HPLC-tandem MS analysis (MRM mode) ------------------------**S18**


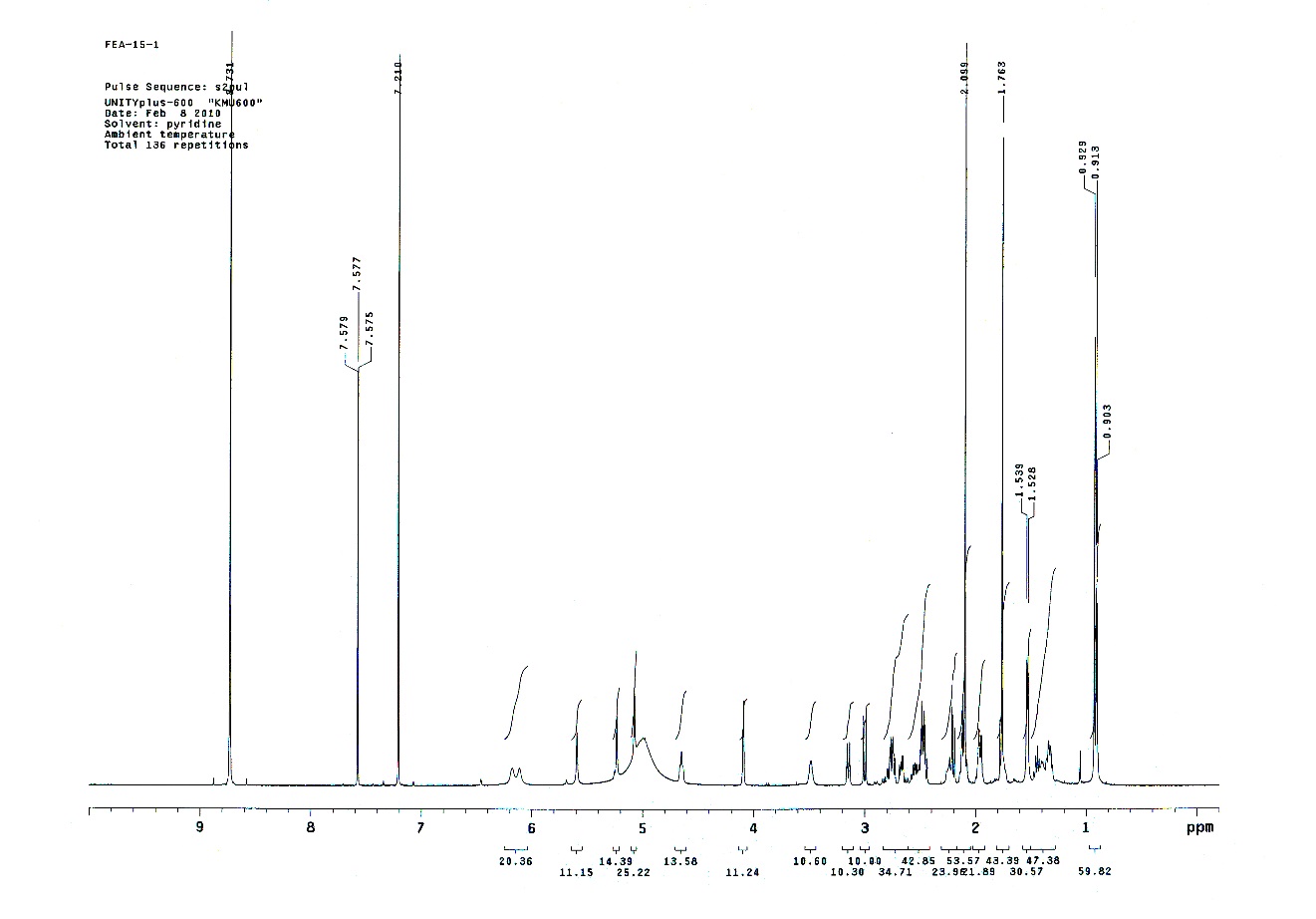


25*S*/*R*-antcin K (**E1/E2**)


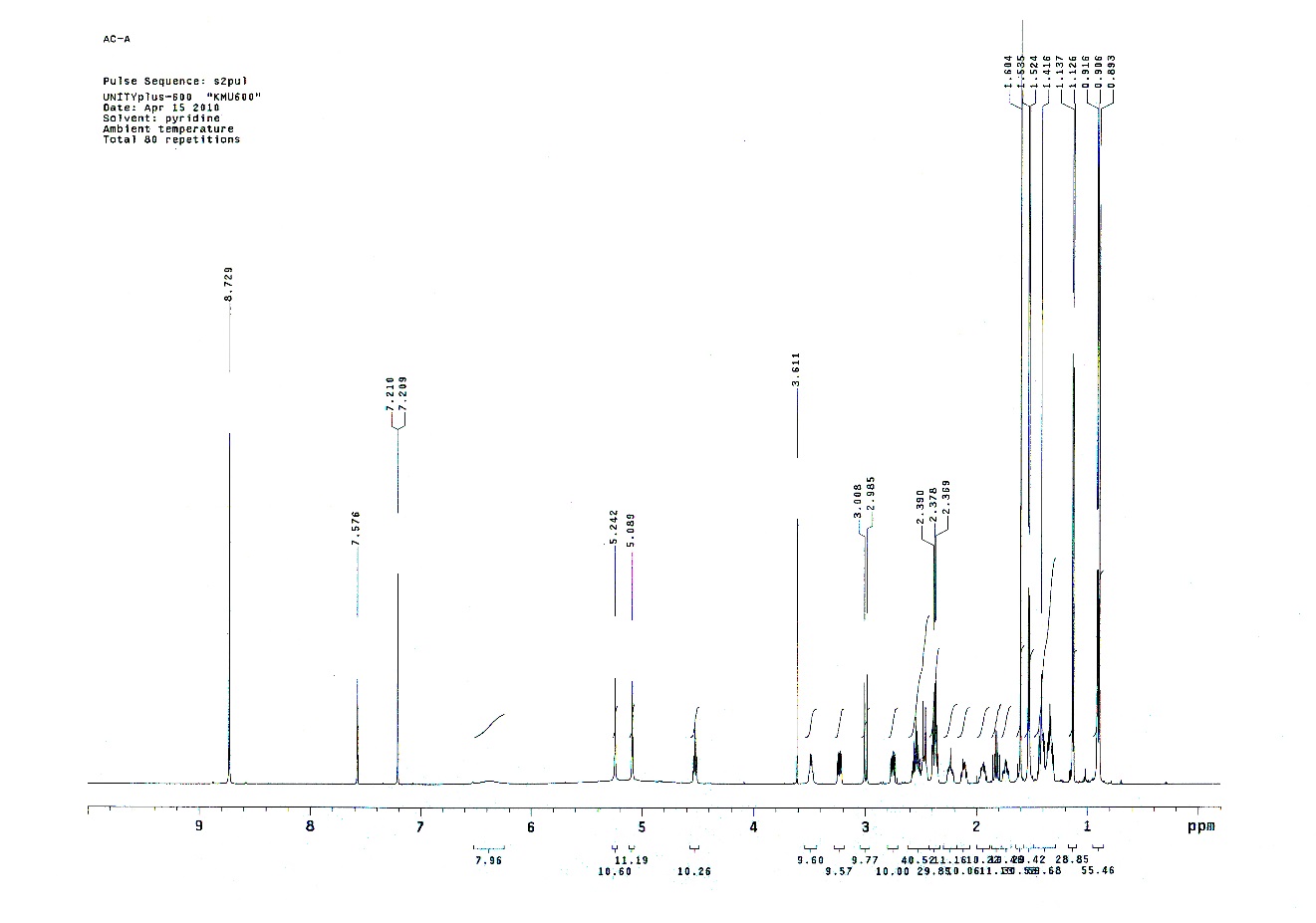


25*S*/*R*-antcin C (**E3/E4**)


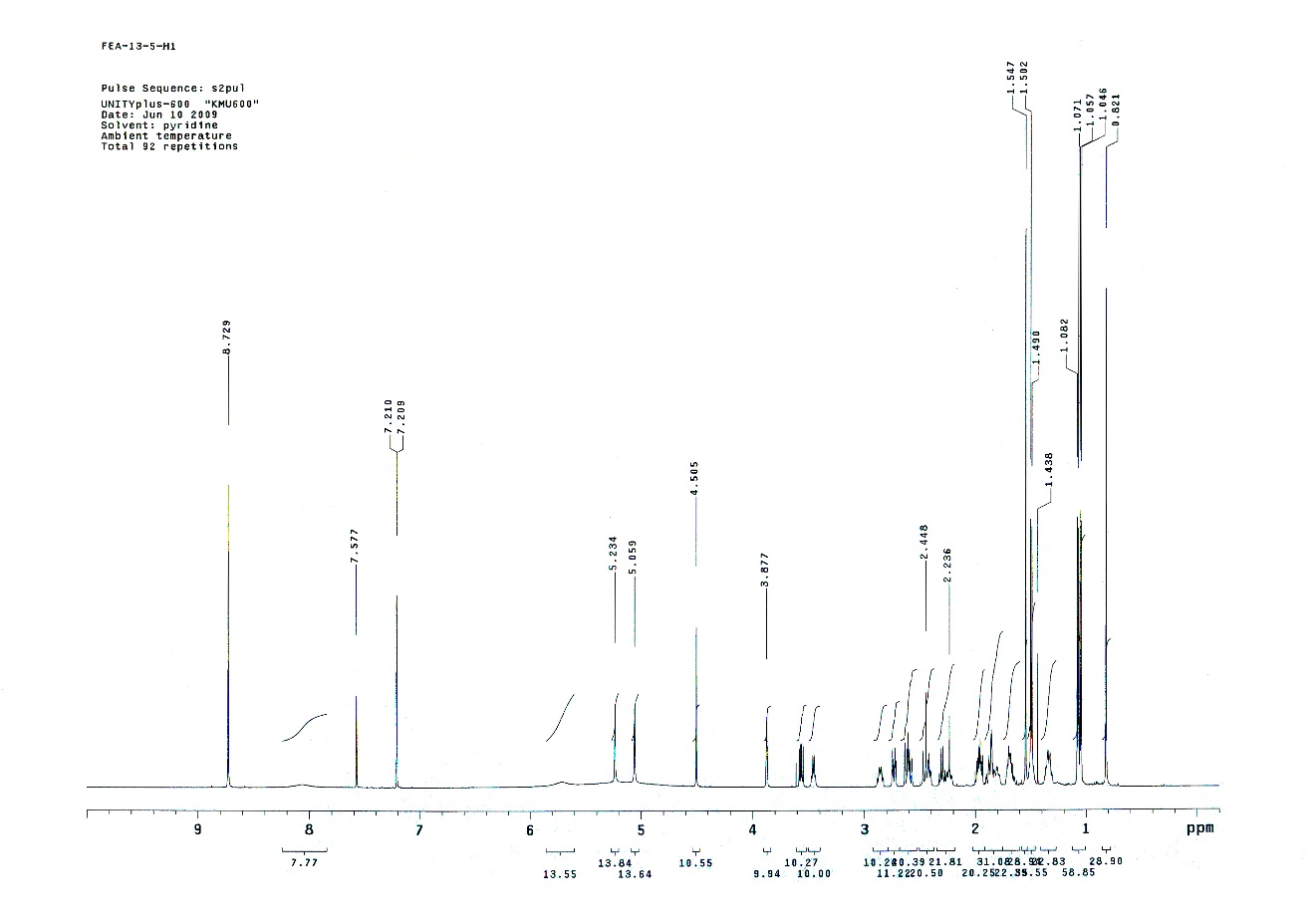


25*R*/*S*- zhankuic acid C (**E5/E6**)


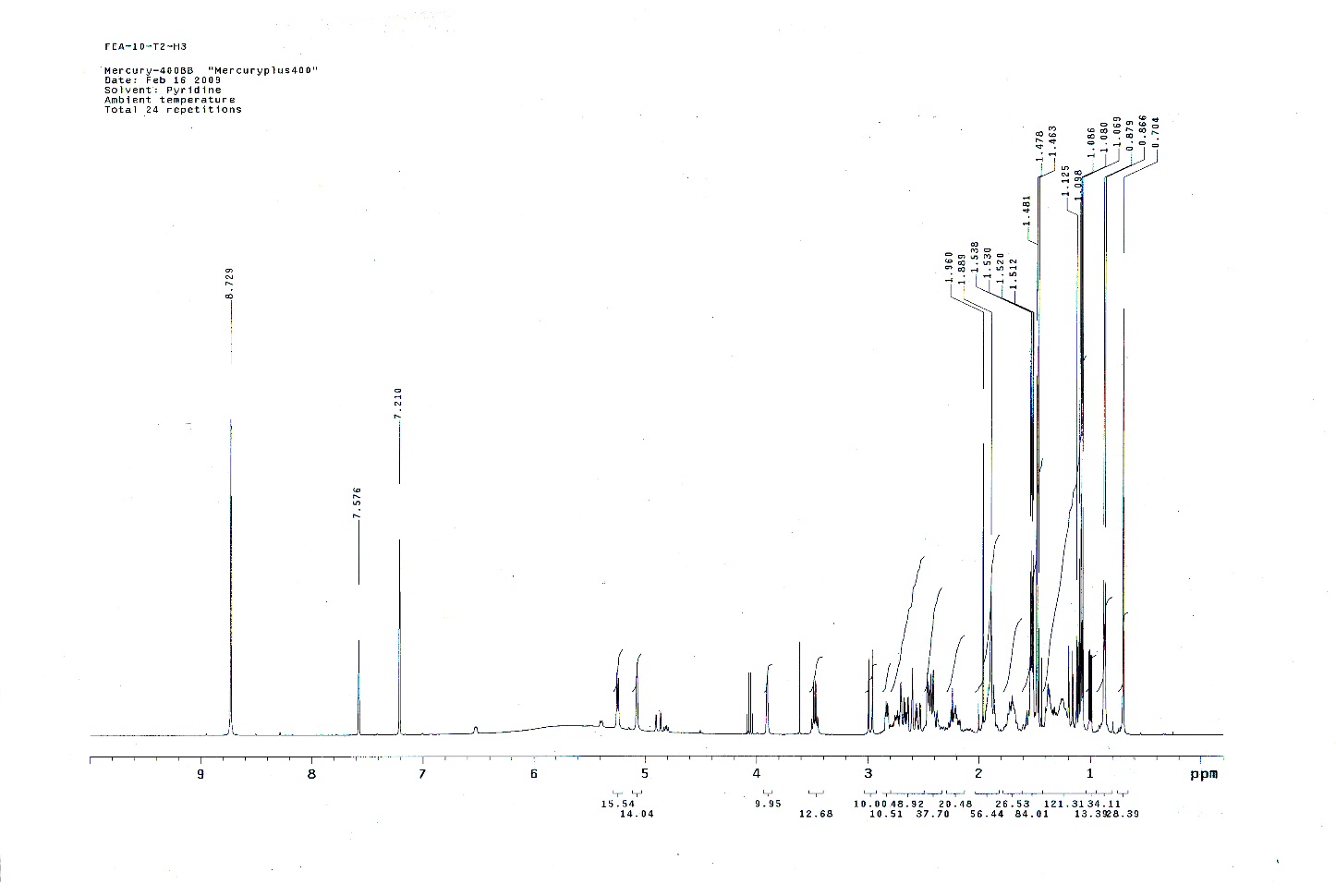


25*R*/*S*- zhankuic acid B (**E7/E8**)


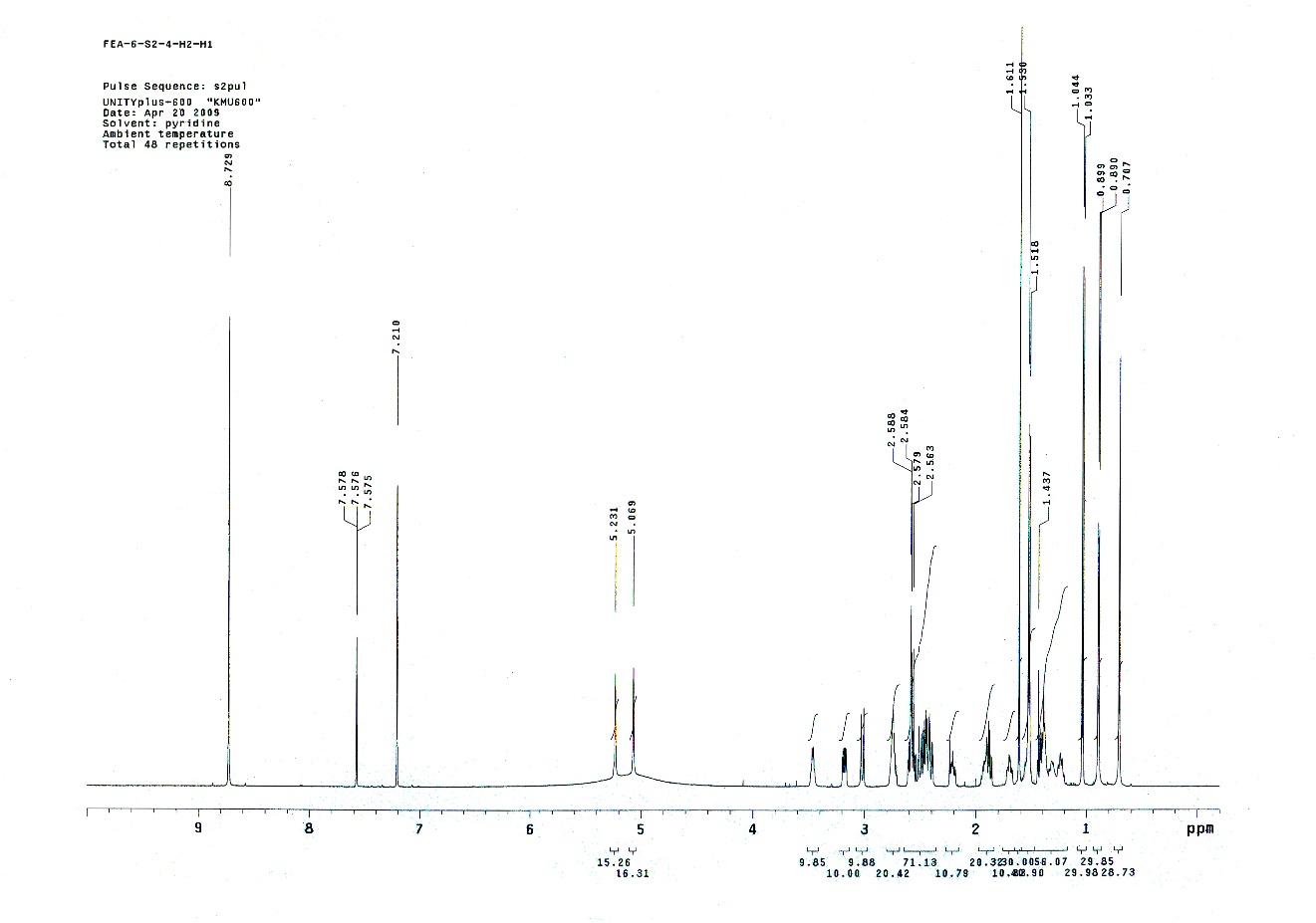


25*S*/*R*- zhankuic acid A (**E9/E10**)


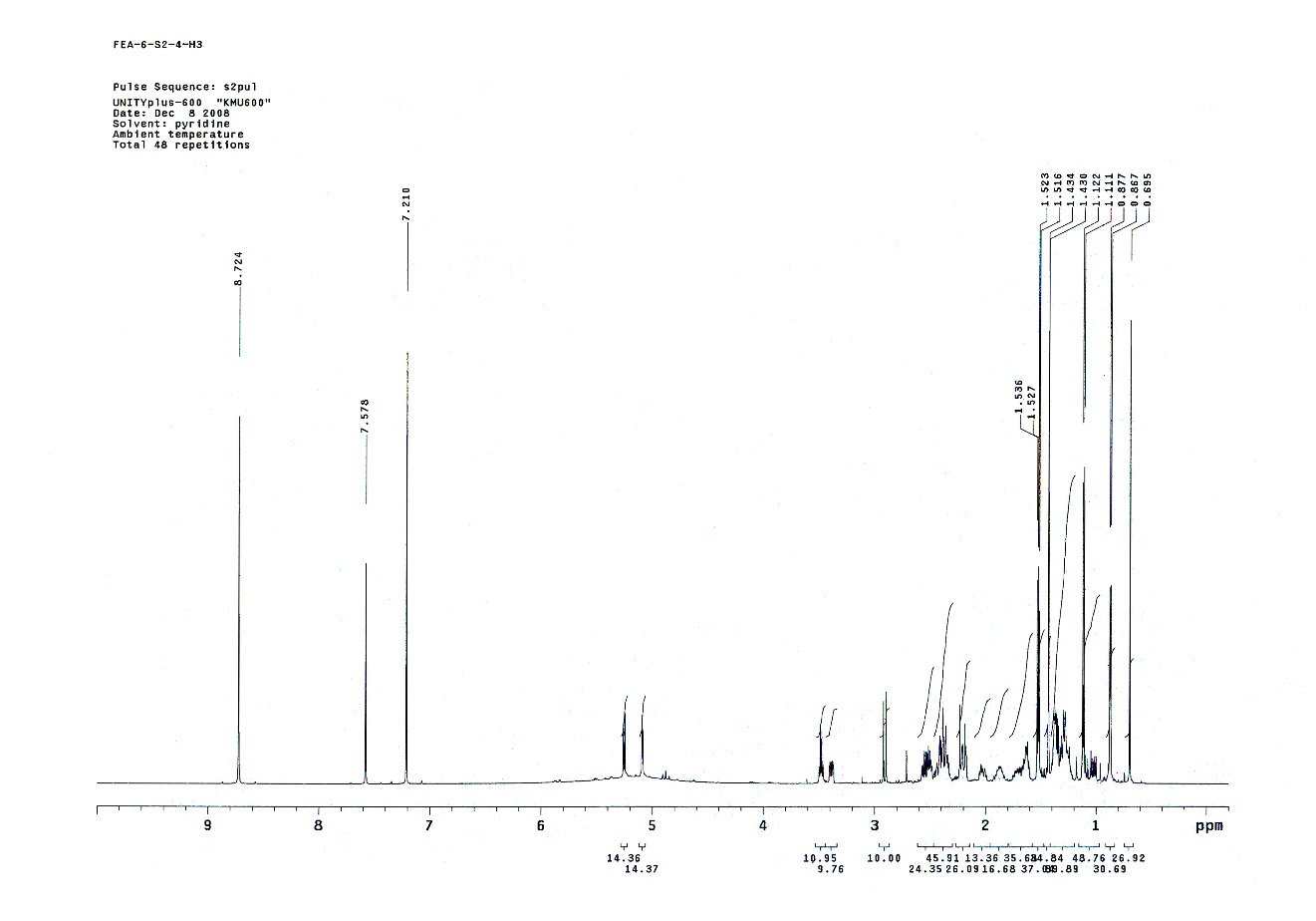


25*S*/*R*-antcin A (**E11/E12**)


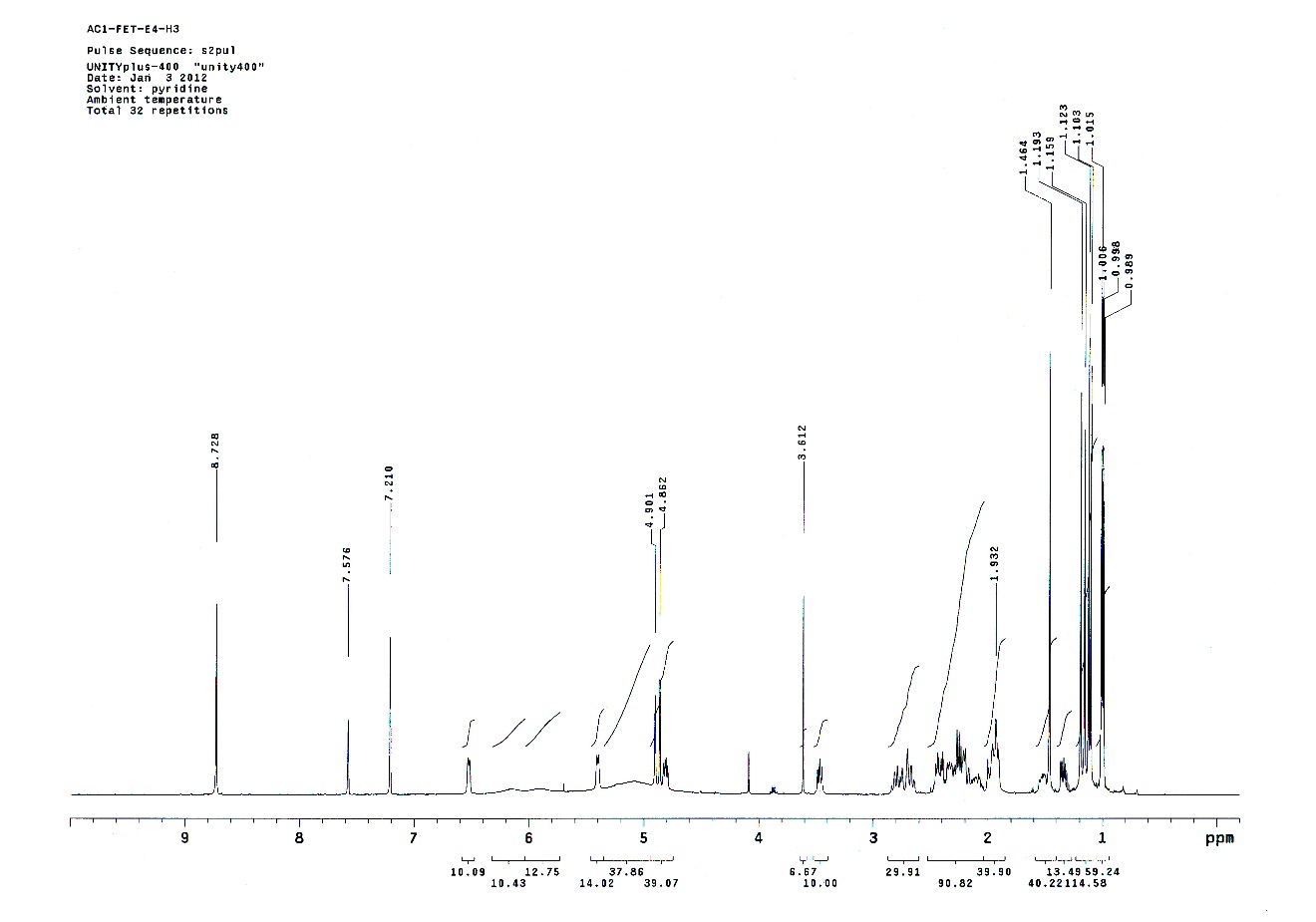


Dehydrosulphurenic acid (**L1**)


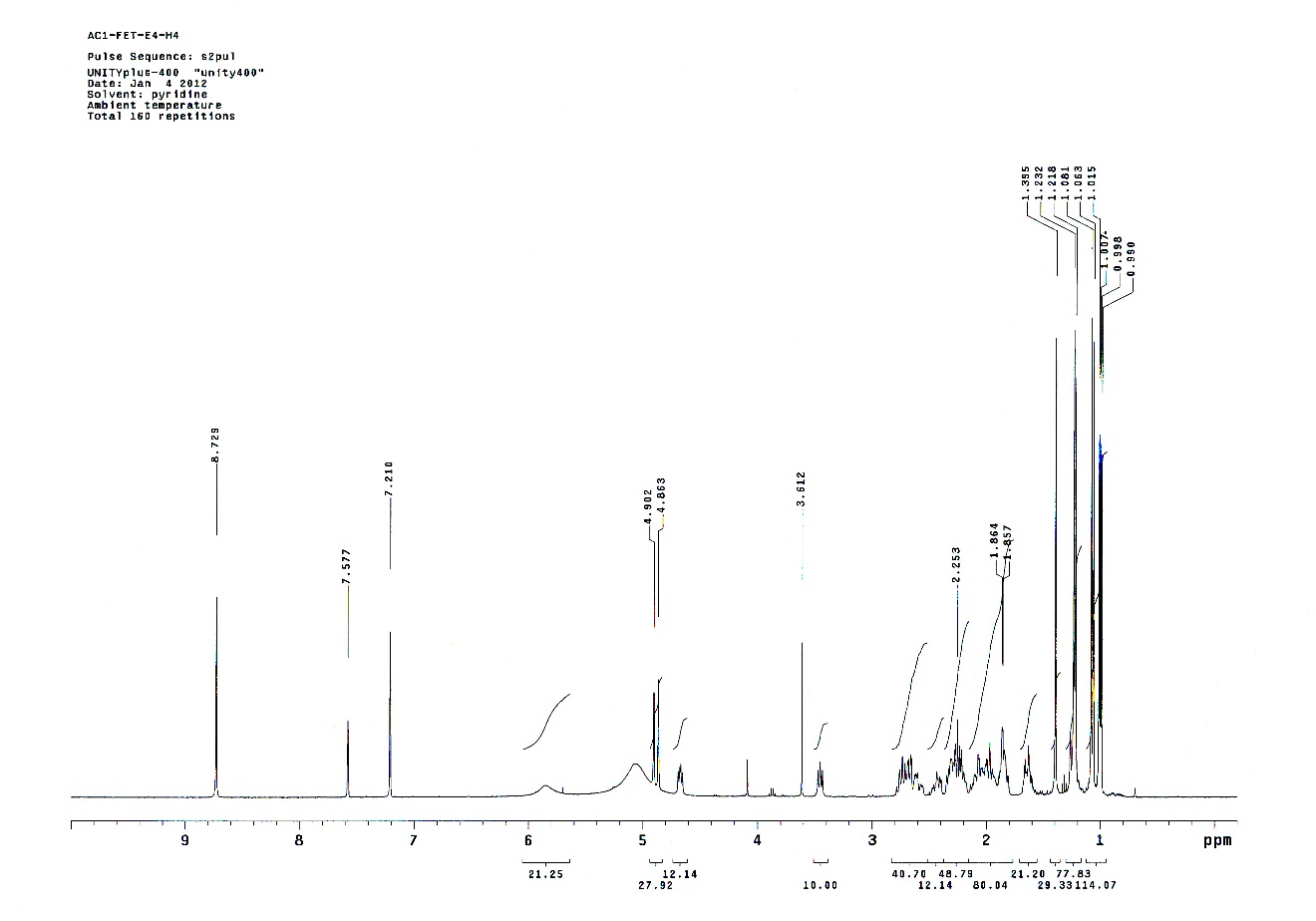


Sulphurenic acid (**L2**)


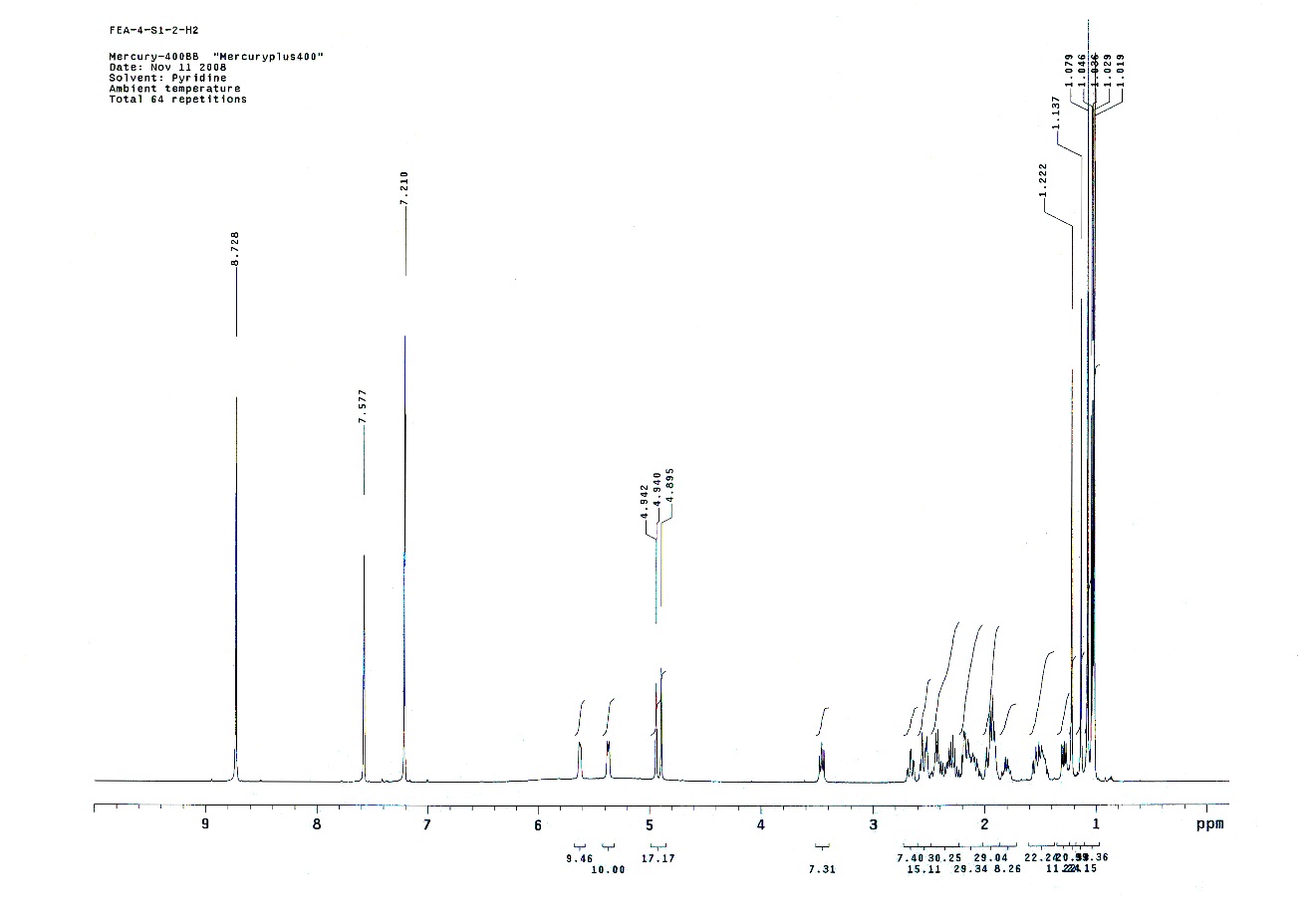


dehydroeburicoic acid (**L3**)


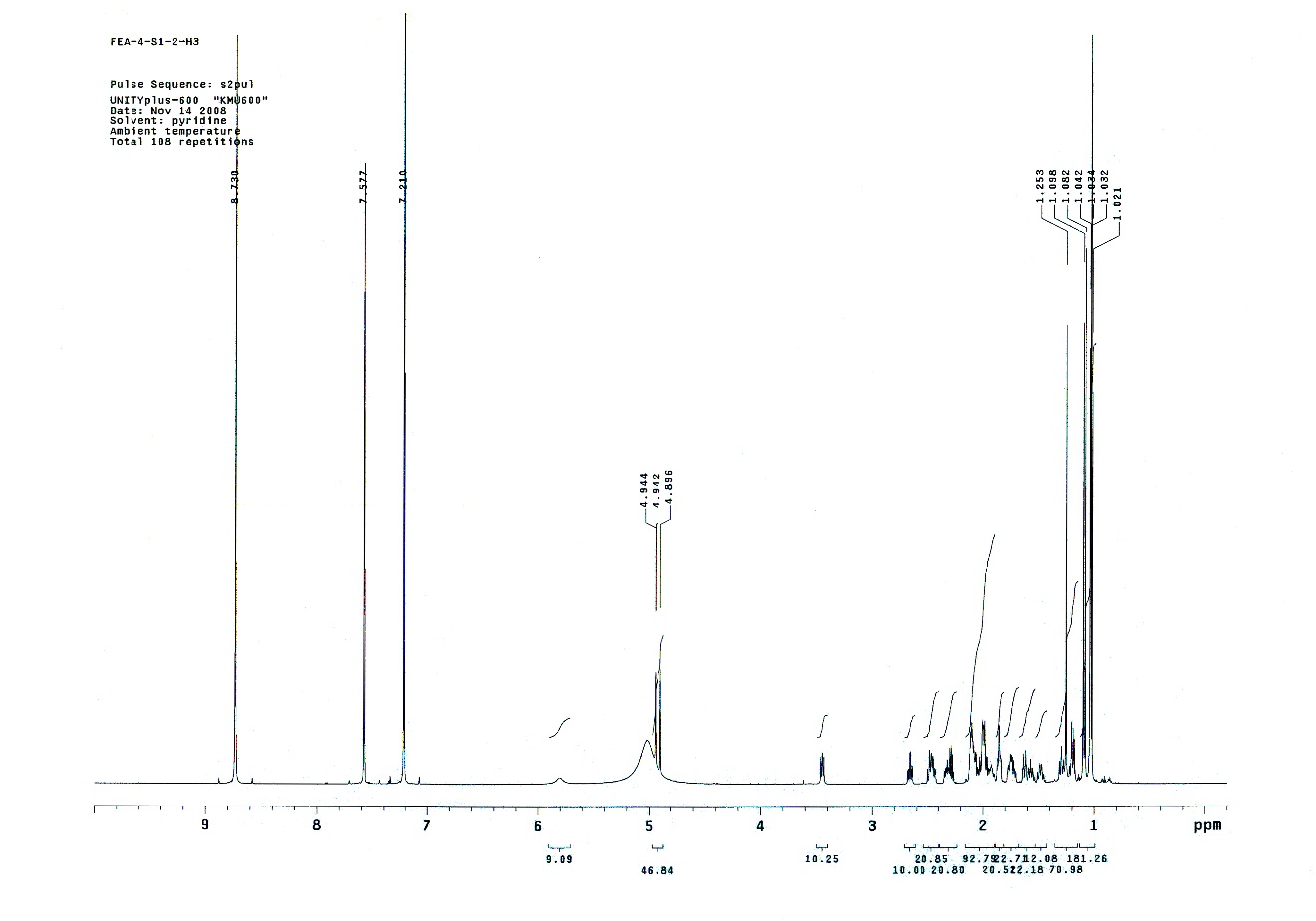


eburicoic acid (**L4**)

**Figure S1.** The ^1^H-NMR spectrum (400 MHz) of the major characteristic ergostane and lanostane triterpenoids (standard compounds used in this study) measured in C_5_D_5_N.


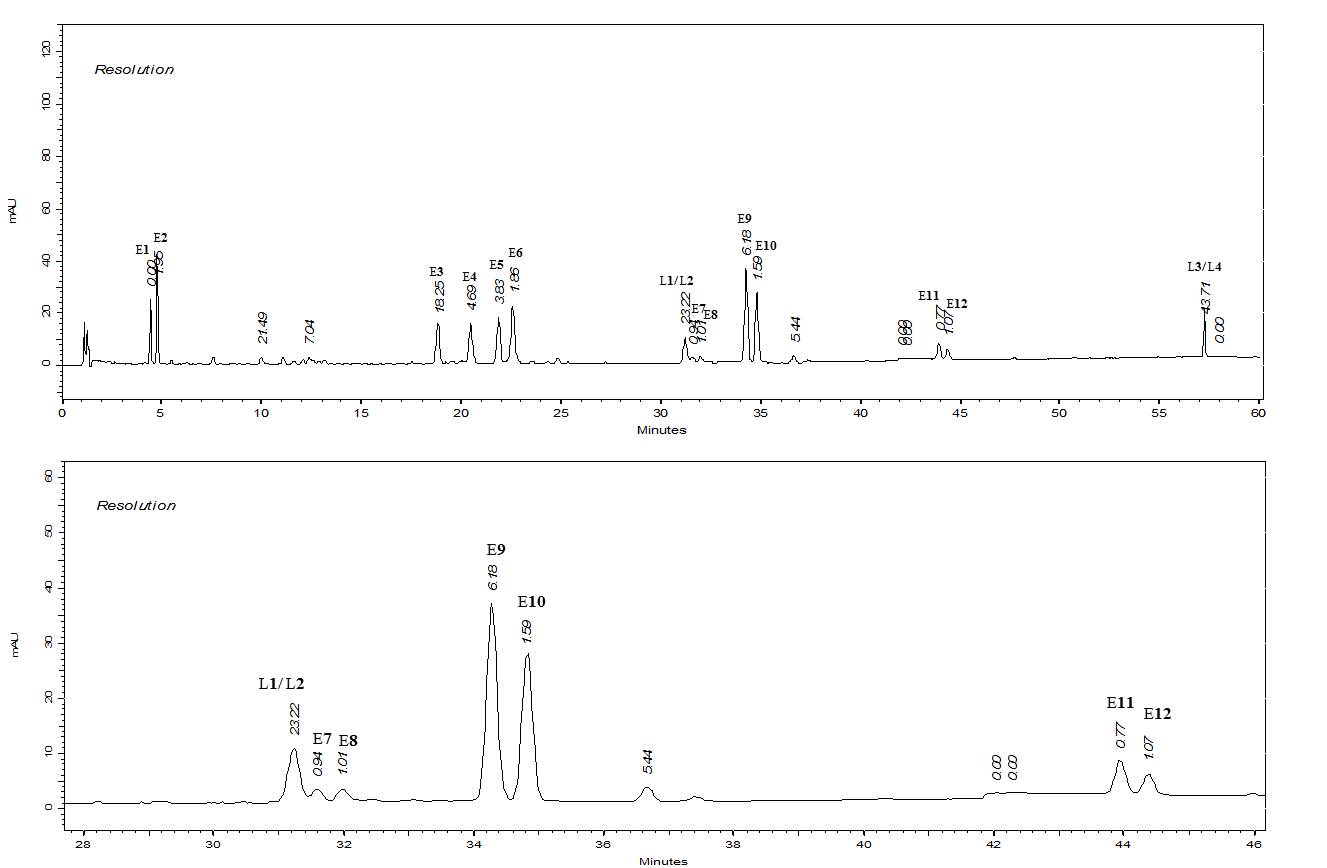


**Figure S2.** An optimum condition and best resolution for separating the major ergostane compounds (**E1**-**E12**) in HPLC analysis and the expanded chromatogram from 28 – 46 min.

25*S*/*R*-antcin K (**E1/E2**)

-18

-18

-44

-44

25*S*/*R*-antcin C (**E3/E4**)

-44

-18

-28

-30

25*R*/*S*- zhankuic acid C (**E5/E6**)

-44

-60

25*R*/*S*- zhankuic acid B (**E7/E8**)

-60

-44

25*S*/*R*- zhankuic acid A (**E9/E10**)

25*S*/*R*-antcin A (**E11/E12**)

-44

-60

Dehydrosulphurenic acid (**L1**)

Sulphurenic acid (**L2**)

dehydroeburicoic acid (**L3**)

eburicoic acid (**L4**)

**Figure S3.** The negative ESI-MS/MS spectra of 25*R*/*S*-ergostane epimers (**E1-E12**) and lanostanes (**L1-L4**).

**Table S1.** Precision and accuracy in determination of ergostane triterpenoids (**E1**-**E12**) using HPLC-tandem MS analysis (MRM mode).

| Analytes | Conc. (ng/mL) | Intra-day (n=5) | | | Inter-day (n=5) | | |
| --- | --- | --- | --- | --- | --- | --- | --- |
|  |  | Conc. found (ng/mL) | RSD  (%)^a^ | RE (%)^a^ | Conc. found (ng/mL) | RSD  (%)^a^ | RE (%)^a^ |
| **E1**  **(**487/443**)** | 50 | 51.63±0.59^b^ | 1.14 | 3.26 | 51.42±1.91 | 3.71 | 2.84 |
|  | 500 | 526.66±15.46 | 2.94 | 5.33 | 487.26±15.97 | 3.28 | -2.55 |
|  | 1000 | 1001.13±8.83 | 0.88 | 0.11 | 1001.61±11.39 | 1.14 | 0.16 |
| **E2**  **(**487/443**)** | 50 | 49.33±2.59 | 5.25 | -1.34 | 51.25±3.10 | 6.05 | 2.50 |
|  | 500 | 525.51±10.39 | 1.98 | 5.10 | 495.60±13.85 | 2.79 | -0.88 |
|  | 1000 | 1000.38±6.78 | 6.75 | 0.04 | 1004.33±7.68 | 0.76 | 0.43 |
| **E3**  **(**469/425**)** | 50 | 49.18±2.34 | 4.76 | -1.64 | 51.20±1.44 | 2.81 | 2.40 |
|  | 500 | 527.88±18.52 | 3.51 | 5.58 | 490.42±29.84 | 6.08 | -1.92 |
|  | 1000 | 1015.26±15.99 | 1.57 | 1.53 | 1010.96±10.04 | 0.99 | 1.10 |
| **E4**  **(**469/425**)** | 50 | 48.04±2.30 | 4.79 | -3.92 | 52.48±2.29 | 4.36 | 4.96 |
|  | 500 | 503.76±6.05 | 1.20 | 0.75 | 495.19±25.56 | 5.16 | -0.96 |
|  | 1000 | 1005.11±3.52 | 0.35 | 0.51 | 1015.64±7.86 | 0.77 | 1.56 |
| **E5**  **(**485/441**)** | 50 | 51.92±0.77 | 1.48 | 3.84 | 48.99±3.79 | 7.74 | -2.02 |
|  | 500 | 508.83±16.99 | 3.34 | 1.77 | 495.08±22.16 | 4.48 | -0.98 |
|  | 1000 | 996.00±7.45 | 0.75 | -0.40 | 1008.16±12.86 | 1.28 | 0.82 |
| **E6**  **(**485/441**)** | 50 | 48.52±1.37 | 2.82 | -2.96 | 52.14±4.61 | 8.84 | 4.28 |
|  | 500 | 500.11±3.97 | 0.79 | 0.02 | 494.55±18.84 | 3.81 | -1.09 |
|  | 1000 | 999.34±4.97 | 0.50 | -0.07 | 1021.63±24.64 | 2.41 | 2.16 |
| **E7**  **(**469/425**)** | 50 | 49.81±0.81 | 1.63 | -0.38 | 51.65±2.45 | 4.74 | 3.30 |
|  | 500 | 503.02±7.94 | 1.58 | 0.60 | 486.92±29.60 | 6.08 | -2.62 |
|  | 1000 | 1006.61±12.65 | 1.26 | 0.66 | 1009.70±20.34 | 2.01 | 0.97 |
| **E8**  **(**469/425**)** | 50 | 51.56±1.30 | 2.52 | 3.12 | 53.32±2.40 | 4.50 | 6.64 |
|  | 500 | 500.27±2.75 | 0.55 | 0.05 | 512.16±9.63 | 1.88 | 2.43 |
|  | 1000 | 1015.83±25.26 | 2.49 | 1.58 | 1016.59±21.86 | 2.15 | 1.66 |
| **E9**  **(**467/423**)** | 50 | 48.94±1.00 | 2.04 | -2.12 | 51.36±2.75 | 5.35 | 2.72 |
|  | 500 | 506.54±9.07 | 1.79 | 1.31 | 498.42±16.19 | 3.25 | -0.32 |
|  | 1000 | 999.32±0.78 | 0.08 | -0.07 | 1015.29±12.61 | 1.24 | 1.53 |
| **E10**  **(**467/423**)** | 50 | 52.35±1.35 | 2.58 | 4.70 | 48.69±3.14 | 6.45 | -2.62 |
|  | 500 | 506.08±10.99 | 2.17 | 1.22 | 507.16±19.23 | 3.79 | 1.43 |
|  | 1000 | 994.33±1.90 | 0.19 | -0.57 | 1016.85±13.03 | 1.28 | 1.69 |
| **E11**  **(**453/409**)** | 50 | 52.17±1.79 | 3.43 | 4.34 | 50.06±1.81 | 3.62 | 0.12 |
|  | 500 | 499.50 ±4.22 | 0.84 | -0.10 | 465.41±3.91 | 0.84 | -6.92 |
|  | 1000 | 1011.03 ±5.50 | 0.54 | 1.01 | 1011.78±12.57 | 1.24 | 1.18 |
| **E12**  **(**453/409**)** | 50 | 52.04±0.55 | 1.06 | 4.08 | 54.07±3.28 | 6.07 | 8.14 |
|  | 500 | 494.08±4.27 | 0.86 | -1.18 | 496.75±14.03 | 2.82 | -0.65 |
|  | 1000 | 1012.60±11.79 | 1.16 | 1.26 | 1012.84±5.37 | 0.53 | 1.28 |

^a^ RSD and RE for relative standard derivation and relative error [(value found - value known) / value known], respectively.

^b^: Mean±S.D.

**Table S2.** Precision and accuracy in the determination of lanostane triterpenoids (**L1-L4**) using HPLC-tandem MS analysis (MRM mode).

| Analytes | Conc. (ng/mL) | Intra-day (n=5) | | | Inter-day (n=5) | | |
| --- | --- | --- | --- | --- | --- | --- | --- |
|  |  | Conc. found (ng/mL) | RSD  (%)^a^ | RE (%)^a^ | Conc. found (ng/mL) | RSD  (%)^a^ | RE (%)^a^ |
| **L1**  **(**483/83**)** | 50 | 48.98±0.11 | 0.22 | -2.04 | 50.88±3.07 | 6.03 | 1.76 |
|  | 500 | 502.73±13.14 | 2.61 | 0.55 | 496.56±24.57 | 4.95 | -0.69 |
|  | 1000 | 1003.12±6.84 | 0.68 | 0.31 | 1015.67±32.56 | 3.21 | 1.57 |
| **L2**  **(**485/355**)** | 50 | 49.00±0.29 | 0.59 | -2.00 | 50.54±2.35 | 4.65 | 1.08 |
|  | 500 | 489.78±10.05 | 2.05 | -2.04 | 493.32±16.52 | 3.35 | -1.34 |
|  | 1000 | 1010.42±12.29 | 1.22 | 1.04 | 1022.14±26.37 | 2.58 | 2.21 |
| **L3**  **(**467/337**)** | 50 | 51.75±1.43 | 2.76 | 3.50 | 48.84±3.95 | 8.09 | -2.32 |
|  | 500 | 510.49±4.28 | 0.84 | 2.10 | 521.44±36.21 | 6.94 | 4.29 |
|  | 1000 | 999.15±0.64 | 0.06 | -0.09 | 1021.19±52.25 | 5.12 | 2.12 |
| **L4**  **(**469/339**)** | 50 | 49.28±0.29 | 0.59 | -1.44 | 54.01±4.44 | 8.22 | 8.02 |
|  | 500 | 500.94±3.23 | 0.64 | 0.19 | 486.63±21.48 | 4.41 | -2.67 |
|  | 1000 | 999.64±6.45 | 0.65 | -0.04 | 1008.20±19.33 | 1.92 | 0.82 |

^a^ RSD and RE for relative standard derivation and relative error [(value found - value known) / value known], respectively.

^b^: Mean±S.D.
